# Morpho-physiological and transcriptomic responses of field pennycress to waterlogging

**DOI:** 10.3389/fpls.2024.1478507

**Published:** 2024-12-18

**Authors:** Rachel Combs-Giroir, Manesh B. Shah, Hari B. Chhetri, Mallory Morgan, Erica Teixeira Prates, Alice Townsend, Mary E. Phippen, Winthrop B. Phippen, Daniel A. Jacobson, Andrea R. Gschwend

**Affiliations:** ^1^ Center for Applied Plant Sciences, The Ohio State University, Columbus, OH, United States; ^2^ Department of Horticulture and Crop Science, The Ohio State University, Columbus, OH, United States; ^3^ Biosciences, Oak Ridge National Laboratory, Oak Ridge, TN, United States; ^4^ Bredesen Center for Interdisciplinary Research and Graduate Education, University of Tennessee-Knoxville, Knoxville, TN, United States; ^5^ School of Agriculture, Western Illinois University, Macomb, IL, United States

**Keywords:** *Thlaspi arvense*, transcriptomics, waterlogging, hypoxia, RNA-seq, recovery, root stress, ERF-VII

## Abstract

Field pennycress (*Thlaspi arvense*) is a new biofuel winter annual crop with extreme cold hardiness and a short life cycle, enabling off-season integration into corn and soybean rotations across the U.S. Midwest. Pennycress fields are susceptible to winter snow melt and spring rainfall, leading to waterlogged soils. The objective of this research was to determine the extent to which waterlogging during the reproductive stage affected gene expression, morphology, physiology, recovery, and yield between two pennycress lines (SP32-10 and MN106). In a controlled environment, total pod number, shoot/root dry weight, and total seed count/weight were significantly reduced in SP32-10 in response to waterlogging, whereas primary branch number, shoot dry weight, and single seed weight were significantly reduced in MN106. This indicated waterlogging had a greater negative impact on seed yield in SP32-10 than MN106. We compared the transcriptomic response of SP32-10 and MN106 to determine the gene expression patterns underlying these different responses to seven days of waterlogging. The number of differentially expressed genes (DEGs) between waterlogged and control roots were doubled in MN106 (3,424) compared to SP32-10 (1,767). Functional enrichment analysis of upregulated DEGs revealed Gene Ontology (GO) terms associated with hypoxia and decreased oxygen, with genes in these categories encoding proteins involved in alcoholic fermentation and glycolysis. Additionally, downregulated DEGs revealed GO terms associated with cell wall biogenesis and suberin biosynthesis, indicating suppressed growth and energy conservation. Interestingly, MN106 waterlogged roots exhibited significant stronger regulation of these genes than SP32-10, displaying a more robust transcriptomic response overall. Together, these results reveal the reconfiguration of cellular and metabolic processes in response to the severe energy crisis invoked by waterlogging in pennycress.

## Introduction

Flooding is a serious threat to global agriculture, causing drastic economic losses from reduced crop production and quality (Rosenzweig et al., 2002; Mirza, 2011). Global warming has led to the increased frequency of heavy precipitation and flooding events. A study of flooding events in the U.S. Midwest between 1948-2012 revealed a positive trend in frequency of heavy rainfall events (Mallakpour and Villarini, 2015). Flooding can negatively impact crop productivity. A U.S. Midwest flooding event in 2019 delayed spring planting for many farmers, impacting crop phenology and yield (Shirzaei et al., 2021). In years with excessive spring rainfall some parts of the U.S. Midwest experience up to 10% yield loss for corn and soybean (Urban et al., 2015). Spring flooding events can also be very impactful on winter annual crops, which are in the reproductive stages in spring. Four winter annual crops that were waterlogged for 14 days at the reproductive stage had significantly decreased yield; Wheat (29%), barley (68%), rapeseed (74%), and field pea (94%) all had significant reductions in seed production per plant (Ploschuk et al., 2020). Studies using global climate modeling data project precipitation increases, up to 30%, in the spring and winter months across the U.S. Midwest and Great Lakes regions in the 2080s, resulting in increased soil moisture (Demaria et al., 2016; Byun and Hamlet, 2018; Byun et al., 2019), and statistical modeling predicts continued increases in flood events throughout the 21^st^ century (Mallakpour and Villarini, 2015; Neri et al., 2020). The risk of crop and yield loss from highly wet springs within the U.S. corn belt region is evident, and crops growing during those months are vulnerable.

Waterlogging occurs when the root zone of a plant becomes saturated or flooded, leading to a low-oxygen, or hypoxic, environment where the diffusion of oxygen and other gases is restricted (Bailey-Serres et al., 2012). This inhibits oxidative phosphorylation, leading to low levels of ATP production in the roots. In most plants, waterlogging typically results in reduced stomatal conductance, root hydraulic conductivity, and nutrient and water uptake (Striker, 2012; Liu et al., 2020), as well as an increase in toxic metabolites and reactive oxygen species (ROS) (Perata and Alpi, 1991; Gibbs and Greenway, 2003; Pucciariello and Perata, 2017). These effects can lead to chlorophyll degradation, leaf chlorosis, and early senescence, resulting in reduced photosynthetic rate and eventually plant death (Ashraf and Mehmood, 1990; Ahmed et al., 2012; Liu et al., 2012; Striker, 2012; Xu et al., 2019). To combat the severe energy shortage under hypoxia, plants will upregulate glycolysis, fermentation, and sugar metabolic processes (Sairam et al., 2008; van Dongen and Licausi, 2015).

Field pennycress (*Thlaspi arvense* L.) is a winter annual oilseed crop native to Eurasia and distributed across temperate regions in North America (Sedbrook et al., 2014). Pennycress is a promising bioenergy crop for the U.S. Midwest Corn Belt due to its high seed oil content and fatty acid oil composition that meets the U.S. renewable fuels standards when converted to biofuel (Moser et al., 2009; Moser, 2012). The extreme cold hardiness and short life cycle of pennycress allow off-season integration into corn and soybean rotations, preventing displacement of summer commodity crops (Fan et al., 2013; Sedbrook et al., 2014). As a winter annual crop, pennycress has potential to prevent nutrient leaching, soil erosion, and spring weed growth, while also providing a food source for native pollinators (Eberle et al., 2015; Johnson et al., 2017; Weyers et al., 2019). These economic and ecological benefits have led to the domestication of pennycress for improved yield, agronomic traits, and oil/protein quality, resulting in a cash crop that has the potential to produce up to 1 billion liters of seed oil annually by 2030 (Phippen et al., 2022). Pennycress is typically planted in autumn (September-October), flowers in early April, and matures in mid to late May meaning that pennycress plants are typically in the reproductive stages when heavy spring precipitation events occur. Anecdotal evidence suggests pennycress is susceptible to damage in areas of fields with standing water, though this has not been documented in controlled experimental investigations to date. For pennycress to become a successful cash cover crop, improved resiliency to abiotic threats faced during the growing season will be essential.

Pennycress research is bolstered by a vast molecular toolkit consisting of a reference genome and transcriptome for the MN106 winter annual line (Dorn et al., 2013, 2015; Nunn et al., 2022), an EMS mutant gene index (Chopra et al., 2018), a sequenced genome of an inbred spring annual line called Spring 32-10/SP32-10 (McGinn et al., 2019), and optimized protocols for transformation and gene editing (McGinn et al., 2019). Pennycress is grouped into lineage 2 of the *Brassicaceae* family with *Brassica* spp. and is closely related to lineage 1 plants, such as *Arabidopsis thaliana* and *Camelina sativa* (Best and Mcintyre, 1975; Dorn et al., 2013). Thus, the abundant data and resources available for *Arabidopsis* and related oilseed crops are easily translatable to pennycress, especially due to a general one-to-one correspondence between *Arabidopsis* genes and pennycress orthologs (Chopra et al., 2018).

Waterlogging and submergence stress have been well studied in model species *A. thaliana* and *Brassica napus* (rapeseed), but is lacking for other *Brassicaceae* crops such as *Brassica rapa, Brassica oleraceae*, *Camelina sativa*, and pennycress (Combs-Giroir and Gschwend, 2024). Furthermore, transcriptomic studies of *Brassicaceae* crops under waterlogging stress at reproductive stages are limited, making it difficult to predict what genes and pathways might be activated in pennycress under spring waterlogging stress, and what changes in gene expression may contribute to improved waterlogging tolerance. The aim of our study was to analyze differences in morpho-physiological and transcriptomic responses between two pennycress lines under reproductive-stage waterlogging stress. The differences observed in morphology, physiology, and yield between the two accessions under 7 days of waterlogging stress provided the opportunity to compare the transcriptomic responses between an accession that had yield significantly impacted by waterlogging (SP32-10) compared to one that did not (MN106). In addition to root tissues, we examined the transcriptomic responses in leaf and seed tissues, which are often overlooked in waterlogging studies despite the economic significance of the seed oil. This study revealed the physiological responses of pennycress to waterlogging and provided insight into the genetic regulation leading to improved pennycress waterlogging tolerance.

## Materials and methods

### Plant material and growth conditions

The pennycress accession MN106 (winter-type) originates from Coates, Minnesota (ABRC stock: CS29149) and is the source of the reference genome, and SP32-10 (spring-type) originates from Bozeman, Montana (ABRC stock: CS29065) and is a rapid-cycling, sequenced lab line. MN106 and SP32-10 seeds were sterilized with a 70% ethanol rinse, followed by 10-minute incubation in 30% bleach/0.01% SDS solution, and then rinsed 3 times with sterile water. Seeds were then plated on petri dishes between two pieces of Whatman paper with 2 mL sterile water, stratified at 4°C for 5 days, and moved to a growth chamber at 21°C with periods of 16-hour light/8-hour dark. Following 1 week of germination, the MN106 seedlings were vernalized at 4°C under 14 hrs of daylight for 3 weeks to induce flowering. Approximately 2 weeks after germination or vernalization, the seedlings were transplanted into plug cells in Berger BM2 Germination Mix (Hummert International, Earth City, MO, United States) and then into 10-cm pots after the first true leaves fully developed. The plants were grown at ~21°C with periods of 16-hour light/8-hour dark either in a growth chamber or a greenhouse. The photosynthetically available radiation (PAR) in the greenhouse was approximately 250 µmol/m^2^/s and in the growth chamber it was approximately 100 µmol/m^2^/s. The media of the pots was 50% Turface MVP calcined clay (PROFILE Products LLC, Buffalo Grove, IL United States), 25% PRO-MIX BX MYCORRHIZAE (Premier Horticulture, Quakertown, PA, United States), and 25% Miracle-Gro All Purpose Potting mix (Scotts Miracle-Gro, Marysville, OH, United States). Plants were watered with Jack’s Nutrients 12-4-16 RO (jacksnutrients.com) at 100 ppm. The water used for watering the plants at the sensitive young seedling through rosette stages was dechlorinated with 2.5 mg/L of sodium thiosulfate pentahydrate.

### Greenhouse experimental design

Following approximately 2 weeks of flowering, during pennycress developmental stage 6 (Verhoff et al., 2022), 18 plants for each accession were placed in 7.6-liter buckets and waterlogged for one week by filling water up to the soil line at the top of the pots. An additional 18 plants for each accession were also placed in buckets but watered normally approximately every other day so that the soil was completely saturated after watering, but dry before the next watering. Our experimental design was the following: six blocks per accession, each block containing two buckets (one waterlogged and one control), each bucket containing three plants of the same accession. The buckets for each accession were randomized on a greenhouse bench in a random complete block design. Since MN106 plants flowered about 1 week later than SP32-10, the waterlogging treatments for each accession took place a week apart. Morphological data was collected immediately after waterlogging where 6 plants per treatment were destructively harvested and measured for plant height, reproductive height (total height-height to first silicle), primary branch number, silicle number, total leaf number, and shoot and root fresh and dry weight. Root and shoot tissues were oven-dried at 50°C for 48 hours. Additionally, 12 plants per treatment/control were allowed to recover and were measured at maturity, when the plants had fully senesced, for plant height, reproductive plant height, silicle number, percentage of aborted silicles, the average number of seeds per silicle for 10 silicles, branch number (total and primary), days until maturity, total dry shoot and root weight, 1000 seed weight, total seed count, total seed weight, and single seed weight.

### Growth chamber experimental design

The greenhouse experiment described above was repeated in a growth chamber using the same growth conditions, in an even ratio of the three soil types, but not watered with Jack’s nutrients. The same experimental design was implemented and there were 12 plants/replicates for each treatment. Immediately after waterlogging, 6 plants per treatment were destructively harvested for RNA-seq (see below). The other 6 plants were monitored daily during maturation until senescence and morphological traits of these plants were measured 1) immediately after waterlogging, 2) after 1 week of recovery, 3) after 2 weeks of recovery, and 4) at maturity when the plants were harvested. The following phenotypes were collected: inflorescence status, silicle number, percentage of aborted and senesced silicles, plant height, reproductive plant height, branch number (total and primary), days until maturity, total dry shoot weight, 1000 seed weight, total seed count, total seed weight, and single seed weight.

### Total seed oil content and fatty acid seed oil composition

Total oil content was determined by nondestructive time-domain nuclear magnetic resonance (TD-NMR) on 450 mg samples of whole pennycress seed and fatty acid seed oil composition was determined by gas chromatography (GC) with 100 mg of whole pennycress seed. The protocols for these procedures can be found in the **Supplementary Methods File**.

### Physiological data collection

During the greenhouse experiment, stomatal conductance (gsw), CO_2_ assimilation (A), and transpiration rate (E) were collected on 12 plants per treatment per accession 1 day before waterlogging, at 7 days of waterlogging, and at 7 days of recovery during the greenhouse experiment using the LI-6800 Portable Photosynthesis System (Licor, Lincoln, NE, USA). Measurements were collected between 10am and 12pm from healthy leaves of roughly the same size on the upper main stem. The instrument flow rate was set to 400 μmol/s, relative humidity at 50%, CO_2__s at 400 ppm, fan speed at 10,000 rpm, and the light source at ambient light conditions. The instrument was stabilized on A, gsw, and fluorescence prior to logging a measurement.

### Statistics

Normality and homogeneity of variance were confirmed using Shapiro-Wilks and Levene’s tests. Traits that did not have equal variances for one or both accessions were plant height, reproductive plant height, shoot fresh weight, percentage of aborted silicles, and thousand seed weight. A Welch’s t-test was used to determine significance between waterlogged and control replicates of each trait for both accessions waterlogged at the reproductive stage. For physiological traits, a repeated measures ANOVA was used with time as a within-subject factor and accession and treatment as between-subject factors to determine the effects of timepoint, accession, treatment, and their interaction. Values for gsw, A, and E were log transformed. Statistically significant main effects were followed by pairwise t-tests with Bonferroni correction for multiple comparisons. All statistical analyses were conducted in RStudio (R version 4.2.1).

### Tissue collection and RNA isolation

Leaves, silicles, and roots were collected from 5 separate plants (replicates) per accession from the growth chamber experiment after 1 week of waterlogging and after 3 hours of recovery. Tissues from control plants were also collected after 1 week, coinciding with the collection of the waterlogged plants. Roots were gently washed in water for ~2 minutes to remove soil and Turface. Tissues were flash-frozen in liquid nitrogen and kept at -80°C until RNA isolation. RNA was isolated from the leaves and roots using the Plant RNeasy Qiagen kit (qiagen.com), and from silicles using the Spectrum Plant Total RNA Kit (sigmaaldrich.com) following kit protocols. RNA samples were stored at -80°C until sent for mRNA library preparation and paired-end Illumina sequencing (NovaSeq 6000 PE150) at Novogene Corporation Inc.

### RNA-seq analysis

Raw sequence read quality was analyzed using FASTQC v0.11.8 (Babraham Bioinformatics, 2018) before and after trimming low-quality reads and adaptor sequences using Trimmomatic v0.38 (Bolger et al., 2014). One sample (MN106 control roots – replicate 1) was removed from the analysis due to poor sequence quality. Clean sequence reads were aligned to the version 2 pennycress genome (GenBank GCA_911865555.2) using STAR v2.7.9a (Dobin et al., 2013). Raw read counts were assigned using featurecounts v2.0.1 (Liao et al., 2014). MultiQC v1.13 was used after FASTQC, STAR, and featurecounts to aggregate output files and obtain a summary of results (Ewels et al., 2016). All processing steps were performed with the Ohio Supercomputer Center (Ohio Supercomputer Center, 1987).

Raw read counts were tested for intersample differential expression (DE) analysis using the DESeq2 v1.38.3 (Love et al., 2014) and “apeglm” to shrink the log2FoldChange (Zhu et al., 2019) in RStudio v4.2.1. The negative binomial GLM was fitted and Wald statistics were calculated, followed by identification of differentially expressed genes using the Benjamini-Hochberg method with a false discovery rate (FDR) < 0.05 and a relative change threshold of 2-fold or greater. A test for interaction of accession and condition was used to reveal genes significantly different between accessions for a given condition or between time points. Volcano plots were generated using the EnhancedVolcano R package v1.16.0 (Blighe et al., 2024). Heatmaps were generated using the pheatmap R package v1.0.12 (Kolde, 2019). A graphic overview of the processing and analysis pipeline, along with all code generated, can be viewed at https://github.com/combsgiroir/Code-for-RNA-seq_Analysis.

### GO and KEGG enrichment analysis

Pennycress genes were assigned an *A. thaliana* gene ID based on shared homology using OrthoFinder v2.5.4 (Emms and Kelly, 2019). Gene Ontology (GO) and Kyoto Encyclopedia of Genes and Genomes (KEGG) enrichment of the RNA-seq results was carried out with the Over-Representation Analysis (ORA) and Gene Set Enrichment Analysis (GSEA) methods using the clusterProfiler R package v4.6.2 and the *A. thaliana* database (Subramanian et al., 2005; Wu et al., 2021; Zhao and Rhee, 2023) with a p-value cutoff < 0.05. Shrunken LFC values were used as input.

### MENTOR algorithm

We identified 2,089 unique significantly differentially expressed genes (DEGs) for control *vs* waterlogged MN106 samples and 432 unique DEGs from control *vs* waterlogging SP32-10 samples (Log_2_FC = ± 1.3). We combined the DEGs for both varieties and used the MENTOR (Multiplex Embedding of Network Topology for Omics Resources) algorithm (Sullivan et al., 2024) to group functionally or mechanistically connected genes into clusters unique to each genotype. In brief, the input for MENTOR is a set of genes that are the seed for a Random Walk with Restart (RWR) algorithm starting from each gene and traversing the network to find functionally (or mechanistically) connected genes based on the network topology. Here, *Arabidopsis thaliana* genes orthologous to *Thlaspi arvense* DEGs were used as starting genes. Random walks traversed an *Arabidopsis thaliana* multiplex network composed of literature curated and exascale/petascale generated gene sets derived from experimental data (Kainer et al., 2024; Sullivan et al., 2024). The output of MENTOR is a dendrogram with clusters of genes ordered by their functional or mechanistic relationships to one another, which was determined by their connectivity among the genes in the multiplex network (Sullivan et al., 2024). A heatmap with up- and downregulated genes for MN106 and SP32-10 in waterlogged relative to control plants is also associated with this dendrogram.

## Results

### Morphology and physiology immediately after waterlogging and during recovery

**Figure 1:**
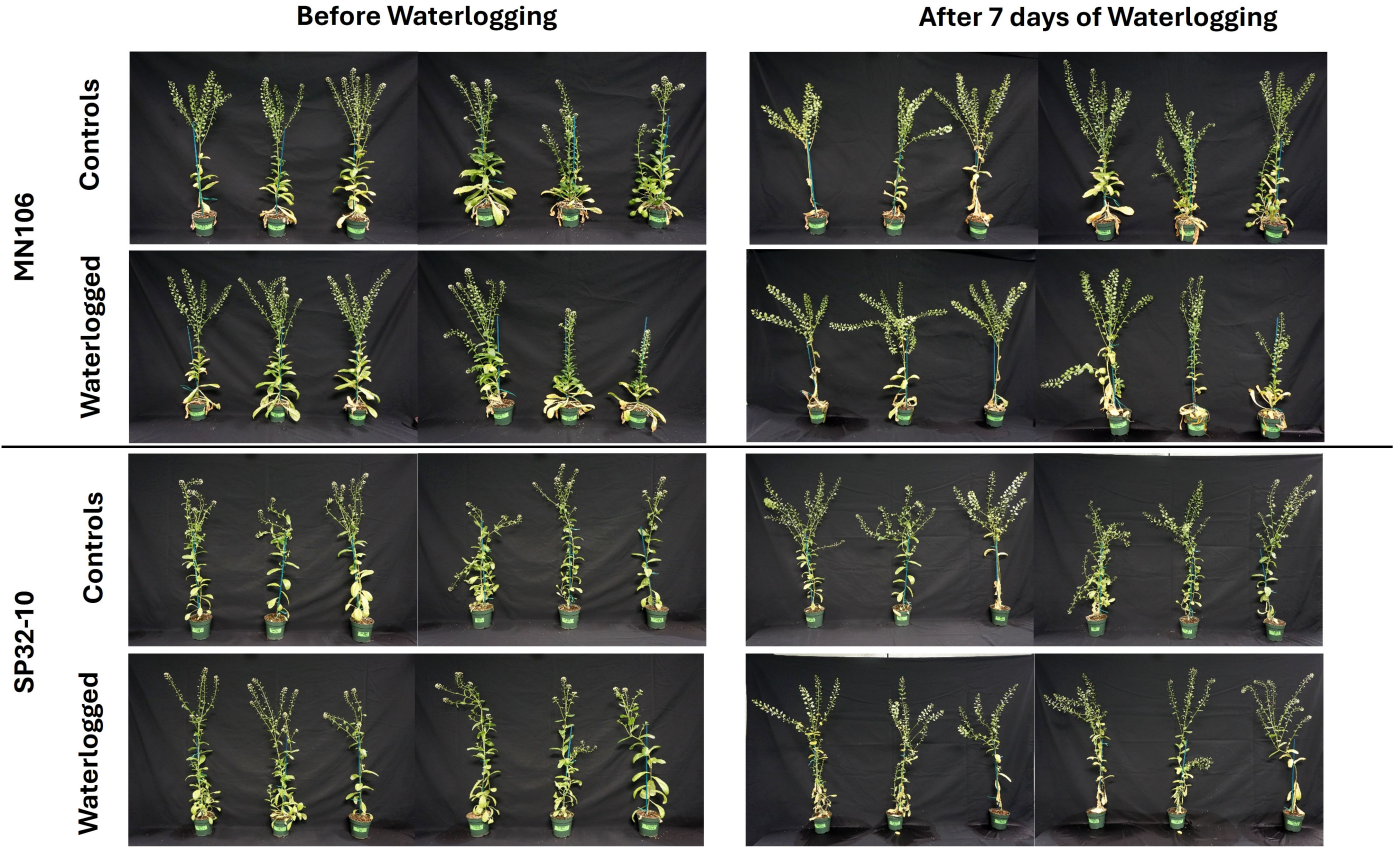
Reproductive-stage pennycress plants before and after 7 days of waterlogging in the greenhouse experiment.

To determine if waterlogging affects pennycress growth, we waterlogged MN106 and SP32-10 accessions for 7 days and recorded morphology and physiology data immediately after waterlogging. These experiments were carried out in a greenhouse and repeated in a growth chamber. The only significant changes to growth and reproduction between control and waterlogged plants immediately following the treatment was a significant reduction (~32%) in leaf number for MN106 waterlogged plants compared to controls (**Figure 1**; **Table 1**; **Supplementary Table 1**) and a significantly higher percentage of dead inflorescences (11%) in SP32-10 waterlogged plants compared to the controls (0%) (**Supplementary Figure 1**; **Supplementary Table 2**). Transpiration rate, stomatal conductance, and CO_2_ assimilation were significantly reduced in both accessions after 7d of waterlogging (**Supplementary Figures 2A–C**). The other traits measured, including shoot and root weight, plant height, and silicle number, were not significantly different between the control and waterlogged plants, suggesting very few observable effects immediately after waterlogging. After a week of recovery, SP32-10 waterlogged plants had significantly more senesced silicles than controls (**Supplementary Figure 3**; **Supplementary Table 3**) and transpiration rate and CO_2_ assimilation remained significantly reduced (**Supplementary Figures 2A–C**), while no significant differences between waterlogged and control plants were observed in MN106 during the recovery period. The SP32-10 waterlogged plants showed early signs of effects on reproductive tissue and maintained effects on plant physiology following a week of recovery.

**Table 1:** Means and standard deviations of morphological traits of waterlogged and control plants immediately after waterlogging in the greenhouse experiment.

	MN106			SP32-10		
	Control	Waterlogged	*P-value*	Control	Waterlogged	*P-value*
Height (cm)	66.5 ± 7.34	68.8 ± 7.73	0.604	61.8 ± 6.84	58.6 ± 5.94	0.412
Reproductive Height (cm)	31.8 ± 7.65	33 ± 8.05	0.802	33.8 ± 6.19	33.3 ± 4.42	0.855
Primary Branch #	11.5 ± 2.59	9.5 ± 1.38	0.135	10.8 ± 0.75	10.2 ± 1.83	0.439
Silicle #	580.7 ± 210.9	498.2 ± 174.6	0.478	297.7 ± 98.6	259.5 ± 63.0	0.446
Leaf #	222.2 ± 49.6	150.2 ± 36.7	0.018*	135.3 ± 23.1	119 ± 29.0	0.308
Root Fresh Weight (g)	6.05 ± 1.75	4.33 ± 1.04	0.072	1.98 ± 0.69	1.57 ± 0.60	0.289
Root Dry Weight (g)	1.03 ± 0.23	0.76 ± 0.22	0.060	0.32 ± 0.081	0.26 ± 0.082	0.242
Shoot Fresh Weight (g)	57.5 ± 16.8	54.1 ± 18.2	0.750	22.7 ± 8.23	19.8 ± 5.30	0.497
Shoot Dry Weight (g)	9.53 ± 1.81	8.64 ± 2.15	0.460	3.76 ± 1.45	3.66 ± 1.16	0.891

Sample size = 6. Asterisk denotes statistical significance of < 0.05. P-values derived from Welch’s t-test between treatments for each accession.

### Morphology, biomass, and yield at the time of harvest

To determine whether one week of waterlogging had lasting effects on pennycress growth and development, morphological traits, biomass, and yield were recorded once the plants had fully matured and senesced (**Table 2**). In SP32-10, waterlogged plants had a significant reduction in the total number of silicles, with an average of about 713 silicles compared to an average of 1,138 silicles in control plants (37.4%). MN106 waterlogged plants also had a reduction in total number of silicles (31.6%), but this was not statistically significant. MN106 waterlogged plants had a significant decrease in mean total primary branches, 11 compared to 16 total primary branches in control plants. Additionally, shoot dry weight of waterlogged plants was significantly reduced by 23.7% in MN106 and 27.3% in SP32-10. The total root dry weight of waterlogged plants was significantly reduced by 41% in SP32-10 and reduced by 30% in MN106, but not significantly. The percentage of aborted silicles was significantly increased by 25% in SP32-10 after waterlogging in the repeated growth chamber experiment (**Supplementary Table 4**).

**Table 2:** Means and standard deviations of morphological traits of waterlogged and control plants at the time of harvest in the greenhouse experiment.

	MN106			SP32-10		
	Control	Waterlogged	*P-value*	Control	Waterlogged	*P-value*
Height (cm)	65.4 ± 6.93	64.2 ± 8.46	0.908	66.7 ± 2.93	66.3 ± 5.34	0.833
Reproductive Height (cm)	29.1 ± 7.19	29.6 ± 9.38	0.875	36.1 ± 4.63	34.8 ± 3.31	0.460
Primary Branch #	15.8 ± 4.85	11.4 ± 3.18	0.018*	10.6 ± 2.61	10.9 ± 3.50	0.794
Branch #	59 ± 37.45	36.7 ± 23.22	0.096	82.4 ± 22.35	65.8 ± 34.60	0.179
Silicle #	911.5 ± 472.5	623.1 ± 232.8	0.076	1138.4 ± 416.8	712.9 ± 318.4	0.011*
Aborted Silicles (%)	42.5 ± 18.8	34.5 ± 27.6	0.198	35.4 ± 11.9	27.9 ± 8.78	0.095
Maturity	48.3 ± 9.22	45.3 ± 11.1	0.931	59.1 ± 4.5	51.9 ± 10.8	0.019*
Root Dry Weight	0.60 ± 0.32	0.42 ± 0.13	0.347	0.61 ± 0.14	0.36 ± 0.16	0.001***
Shoot Dry Weight	15.6 ± 4.26	11.9 ± 4.24	0.046*	13.9 ± 2.69	10.1 ± 3.97	0.013*
Total Seed Count	3235.2 ± 1011.2	2906.1 ± 1142.4	0.462	4546.7 ± 1007.1	3148.9 ± 1148.1	0.003**
Total Seed Weight (g)	3.73 ± 1.45	3.0 ± 1.18	0.186	4.61 ± 1.05	3.16 ± 1.45	0.011*
Thousand Seed Weight (g)	1.15 ± 0.27	1.06 ± 0.39	0.266	1.01 ± 0.05	0.93 ± 0.12	0.143
Single Seed Weight (mg)	0.93 ± 0.093	0.78 ± 0.18	0.019*	1.11 ± 0.15	1.00 ± 0.35	0.344
Seeds per silicle	8.79 ± 1.05	8.72 ± 0.76	0.844	9.43 ± 0.76	8.74 ± 0.83	0.045*
Oil Content ( % DWB)	30.1 ± 2.19	28.1 ± 2.13	0.120	27.7 ± 2.46	26.8 ± 3.70	0.360

Sample size = 12. Asterisk denotes statistical significance of < 0.05. DWB, dry weight basis. P-values derived from Welch’s t-test between treatments for each accession.

Several parameters of seed yield were also measured (**Table 2**). Compared to controls, SP32-10 waterlogged plants had a significant reduction in total seed count by 31%, total seed weight by 31%, and seeds per silicle by 7%. The repeated growth chamber experiment also presented similar results (**Supplementary Table 4**). Waterlogged MN106 plants only had a significant reduction in single seed weight by 16% compared to controls (**Table 2**), which was also observed in the repeated growth chamber experiment (24% reduction) (**Supplementary Table 4**).

**Figure 2:**
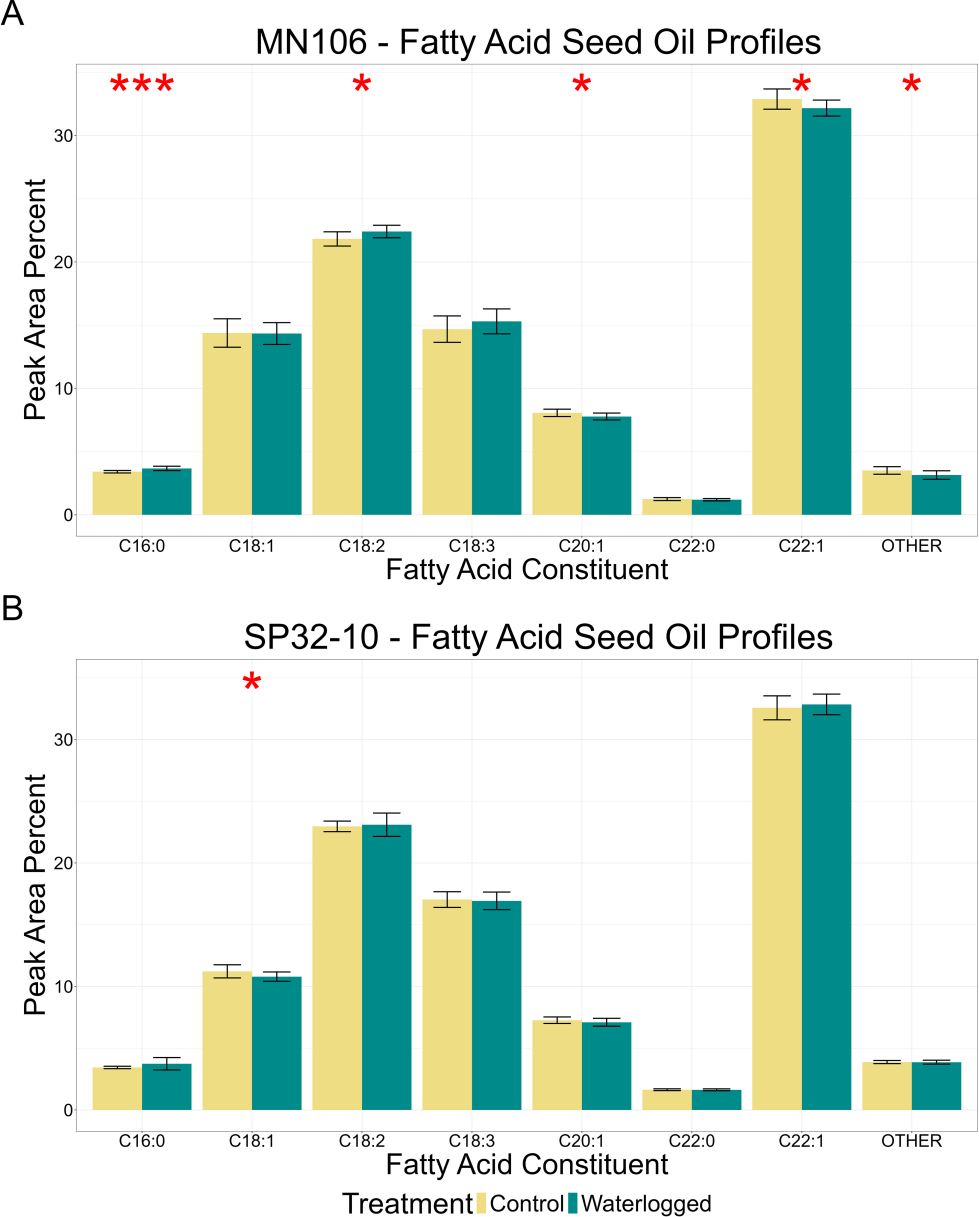
Fatty acid oil profiles of mature seed after waterlogging treatment in the greenhouse experiment in **(A)** MN106 and **(B)** SP32-10. Fatty acid constituents are C16:0 = palmitic acid, C18:1 = oleic acid, C18:2 = linoleic acid, C18:3 = linolenic acid, C20:1 = eicosenoic acid, C22:0 = behenic acid, C22:1 = erucic acid. *p-value < 0.05, ***p-value < 0.001, bars denote standard deviation.

Since pennycress is harvested for seed oil, we wanted to test if waterlogging caused any changes in oil content or fatty acid profiles in mature seeds. In MN106, there was no significant difference in total oil content after waterlogging in the greenhouse experiment (**Table 2**), but there was a significant 10.6% reduction in the repeated growth chamber experiment (**Supplementary Table 4**). In SP32-10, no significant differences were detected in total oil content. MN106 waterlogged seeds showed several significant changes in fatty acid constituents compared to control seeds following the greenhouse experiment, such as an increase in palmitic acid and linoleic acid and decreases in eicosenoic acid, erucic acid, and constituents grouped into the “other” category (**Figure 2A**). Only a significant increase in behenic acid in MN106 was observed in the repeated growth chamber experiment (**Supplementary Figure 4**). SP32-10 waterlogged seeds only showed a significant decrease in oleic acid (**Figure 2B**). At the time of harvest, after pennycress had recovered from 7 days of waterlogging and senesced, while significant differences in growth, yield, and oil quality traits were detected in both accessions, only SP32-10 had a significant reduction in seed yield traits. Therefore, to discern the genetics underlying these phenotypic differences, we explored the transcriptomic response to 7d of waterlogging between the two accessions.

### Differentially expressed genes between waterlogged and control plants (MN-7WL and SP-7WL)

**Figure 3:**
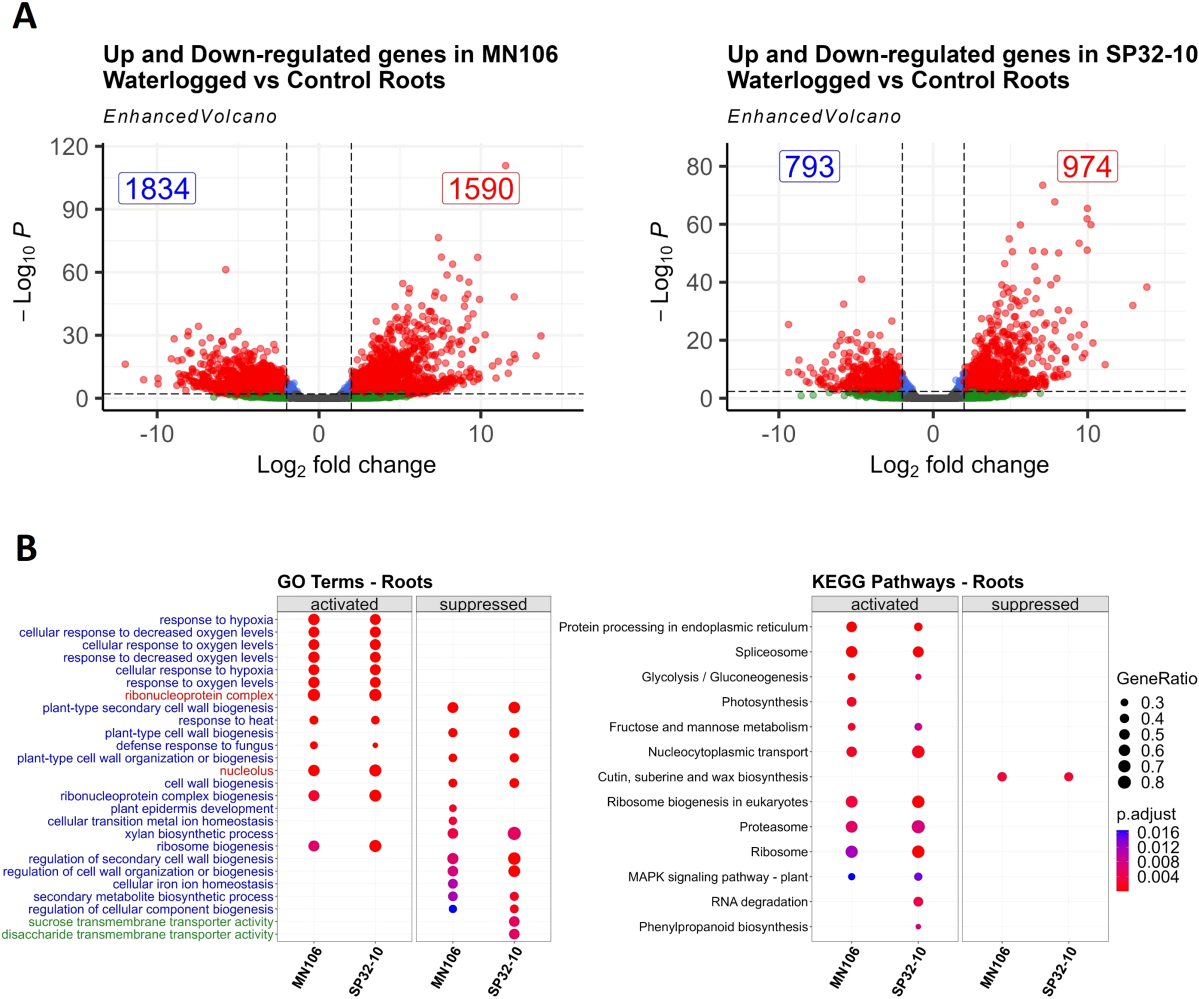
Differentially expressed genes and enrichment results for waterlogged roots compared to controls. **(A)** Volcano plots showing the log2FC of downregulated and upregulated differentially expressed genes (red dots) in waterlogged roots based on cutoffs of |log2FC| ≥ 1 and a p-value ≤ 0.05, **(B)** Dot plot of 10-30 most significant gene ontology and KEGG terms of gene set enrichment analysis in MN-7WL and SP-7WL roots. Blue, red, and green terms represent biological process, cellular component, and molecular function, respectively. GeneRatio represents the ratio of the number of genes from the input data that belong to each term out of the total number of genes in the database associated with that term and p.adjust represents the level of significance of the enrichment.

To determine the genes contributing to a response to waterlogging stress in pennycress, we waterlogged plants for 7 days following 2 weeks of flowering and compared the transcriptomes of the roots, leaves, and silicles to those of plants watered normally for both MN106 (MN-7WL) and SP32-10 (SP-7WL). After 7 days of waterlogging, despite few phenotypic changes in the plants, there was a large transcriptomic response. A total of 3,424 genes (~13% of genes in the genome) were differentially expressed in MN-7WL roots, with 1,590 genes upregulated and 1,834 downregulated, whereas only 1,767 genes (~7% of total genes) were differentially expressed in SP-7WL roots, with 974 genes upregulated and 793 downregulated under waterlogging (**Figure 3A**; **Supplementary Figure 5**; **Supplementary File 1**). Significantly fewer differentially expressed genes (DEGs) were identified between the waterlogged and control pennycress leaves and silicles (**Supplementary Figure 5**). After 7 days of waterlogging, there were only 27 DEGs, 26 upregulated and 1 downregulated, in MN106 leaves, whereas no DEGs were detected in the leaves of SP32-10. There were 37 and 2 DEGs in MN106 and SP32-10 silicles, respectively, all upregulated compared to the controls. These results suggest MN106 had a broader transcriptomic response to waterlogging (more DEGs in more tissues) compared to SP32-10 and roots had the greatest transcriptomic response to waterlogging compared to shoot tissues directly following waterlogging.

Next, we performed a gene set enrichment analysis (GSEA), which factors in the log2FC values of all genes across the transcriptome landscape, as opposed to an ORA which only factors in the DEGs (Subramanian et al., 2005; Zhao and Rhee, 2023). All genes for each comparison were ranked by their log2 fold change (log2FC) values, and the GSEA determined which genes at the top and bottom of the list were enriched for certain GO terms and KEGG pathways. We found 128 enriched GO terms in MN-7WL and 135 in SP-7WL, where 100 had positive enrichment scores (genes were mostly upregulated) and about 30 terms had negative enrichment scores (genes were mostly downregulated) (**Supplementary File 2**; **Figure 3B**). In MN-7WL and SP-7WL roots, upregulated genes were significantly enriched for GO terms related to response to hypoxia/decreased oxygen and ribonucleoprotein complex biogenesis, whereas downregulated genes were significantly enriched for GO terms related to cell wall biogenesis. Interestingly, MN-7WL and SP-7WL had enrichments of downregulated genes in unique GO categories, as well, including those related to metal ion homeostasis for MN-7WL and sugar transmembrane transport activity for SP-7WL. Genes involved in 12 KEGG pathways were enriched in MN-7WL and 14 in SP-7WL, and only 1-2 pathways had downregulated genes (**Supplementary File 3**; **Figure 3B**). Upregulated genes were significantly enriched for KEGG pathways related to the spliceosome/ribosome/proteasome, nucleocytoplasmic transport, fructose and mannose metabolism, and glycolysis/gluconeogenesis, whereas downregulated genes were significantly enriched for cutin, suberin, and wax biosynthesis pathways.

We further investigated the DEGs that contributed to the major GO and KEGG term enrichment categories (**Supplementary File 4**). Some of the upregulated DEGs under waterlogging involved in hypoxia responses in pennycress were involved in signaling low oxygen levels- *HYPOXIA RESPONSIVE ERFs (HRE1* and *HRE2*), sugar metabolism- *SUCROSE SYNTHASE 1 (SUS1)*, glycolysis- *FRUCTOSE-BISPHOSPHATE ALDOLASEs (FBA6*, *FBA3)*, *HEXOKINASE 3 (HXK3)*, *ENOLASE 2 (ENO2)*, *PHOSPHOENOLPYRUVATE CARBOXYKINASE 2* (*PCK2)*, and fermentation- *PRYUVATE DECARBOXYLASE 2 (PDC2)*, *ALCOHOL DEHYDROGENASE 1 (ADH1)*. Furthermore, some of the downregulated DEGs involved in secondary cell wall biogenesis had roles in lignin and cellulose biosynthesis and cell morphology, such as *LACCASE 10 (LAC10)*, *MYB DOMAIN PROTEIN 20 AND 58 (MYB20*, *MYB58)*, *C-TERMINALLY ENCODED PEPTIDE RECEPTOR 1* (*CEPR1*), and *CELLULOSE SYNTHASE A4* (*CESA4*). Genes involved in cutin/suberin/wax biosynthesis were also downregulated, such as *FATTY ACID REDUCTASEs (FAR5*, *FAR4), CYTOCHROME P450 (CYP86)*, *ABERRANT INDUCTION OF TYPE THREE 1* (*ATT1)*, and *CALEOSIN 6 (CLO6)*, which have roles in suberin biosynthesis and fatty acid metabolic processes (**Supplementary File 4**). Furthermore, several of the top 10 upregulated DEGs in waterlogged roots for each accession (**Table 3**) were involved in ethylene synthesis and stress responses, like fermentation, further supporting some of the stress response gene enrichment categories detected with the GSEA analyses.

**Table 3:** List of top significant differentially expressed genes (DEGs) in waterlogged vs control roots for MN106 and SP32-10 with log2FC, significance level (padj), and differential expression rank among the total DEG list.

				MN106 (MN-7WL)			SP32-10 (SP-7WL)		
Pennycress ID	Arabidopsis ID	Gene Name/Putative Function	Abbr.	log2FC	padj	rank^B^	log2FC	padj	rank
TAV2_LOCUS11649	−^A^	*cytochrome P450*	*−*	11.53	3E-107	**1**	7.872	1.3E-64	**3**
TAV2_LOCUS281	AT4G10270	*WOUND-INDUCED POLYPEPTIDE 4*	*WIP4*	7.379	3.1E-73	**2**	7.087	3.3E-70	**2**
TAV2_LOCUS6929	AT2G02010	*GLUTAMATE DECARBOXYLASE 4*	*GAD4*	7.561	3.7E-64	**3**	5.339	1E-31	30
TAV2_LOCUS17926	AT1G67100	*LOB DOMAIN-CONTAINING PROTEIN 40*	*LBD40*	9.802	3.7E-64	**4**	6.581	4.9E-43	16
TAV2_LOCUS19745	AT5G54960	*PYRUVATE DECARBOXYLASE 2*	*PDC2*	8.257	5.3E-61	**5**	8.634	2.8E-75	**1**
TAV2_LOCUS5321	AT1G64600	*RIBOSOMAL PROTEIN MS22*	*MS22*	-5.765	1.8E-58	**6**	-4.637	9.7E-39	18
TAV2_LOCUS23530	AT4G37710	*VQ MOTIF-CONTAINING PROTEIN 29*	*VQ29*	7.909	5.8E-56	**7**	7.198	4.9E-48	13
TAV2_LOCUS17395	AT1G73010	*PHOSPHATE STARVATION-INDUCED GENE 2*	*ATPS2*	8.691	1.6E-54	**8**	3.199	9.1E-06	597
TAV2_LOCUS5907	AT3G29970	B12D protein	*−*	9.259	1.1E-52	**9**	9.984	1.7E-62	**4**
TAV2_LOCUS5664	AT5G26710	Glutamyl/glutaminyl-tRNA synthetase	*−*	5.177	4.3E-52	**10**	4.624	4.9E-44	15
TAV2_LOCUS10091	AT2G19590	*ACC OXIDASE 1*	*ACO1*	8.984	1.6E-41	21	9.962	5.3E-59	**5**
TAV2_LOCUS13607	AT2G29870	Aquaporin-like superfamily protein	*−*	9.174	2.1E-32	47	10.21	4.7E-57	**6**
TAV2_LOCUS18148	AT1G77120	*ALCOHOL DEHYDROGENASE 1*	*ADH1*	5.595	9.9E-50	11	5.641	4.7E-57	**7**
TAV2_LOCUS26083	AT4G20840	*OLIGOGALACTURONIDE OXIDASE 2*	*OGOX2*	4.701	2E-41	22	4.92	2.7E-52	**8**
TAV2_LOCUS21261	−	Unknown - protein kinase domain	*−*	6.627	1.7E-25	103	9.447	7.4E-51	**9**
TAV2_LOCUS10094	−	*ACC OXIDASE 1*	*ACO1*	9.209	7.3E-36	33	9.969	1.7E-48	**10**

^A^Some genes did not have an Arabidopsis ortholog, therefore, the protein sequences were BLASTed to find a putative function based on conserved domains and orthology to genes from other species.

^B^Bolded rank values indicate the top 10 most significantly differentially expressed genes for each accession.

Since several changes in fatty acid constituents were detected in the seed oil of waterlogged plants at maturity, we also used GSEA to examine gene enrichment results across the entire silicle transcriptome in waterlogged compared to control samples. Upregulated genes in MN-7WL silicles were enriched in functions related to fatty acid catabolism and lipid modification, and downregulated genes were enriched for fatty acid biosynthesis (**Supplementary File 2**). Additionally, genes involved in the synthesis of very long-chain fatty acids (VLCFAs) were some of the genes belonging to the enriched fatty acid biosynthesis and fatty acid catabolic categories, such as genes encoding 3-ketoacyl-CoA synthase enzymes, plant natriuretic peptide (PNP), ELO homolog 2, and Acyl-CoA oxidase 1 and 2. Oppositely, SP-7WL silicle genes did not have any GO category enrichments, which is in alignment with the very few differences that were detected in the seed oil profiles after waterlogging. Overall, these root and silicle enrichment results, and the DEGs within the enriched categories, indicate changes in gene expression related to stress responses, metabolism, transcription/translation, and development.

### Differentially expressed genes between accessions after waterlogging (MN-7WL *vs* SP-7WL)

**Figure 4:**
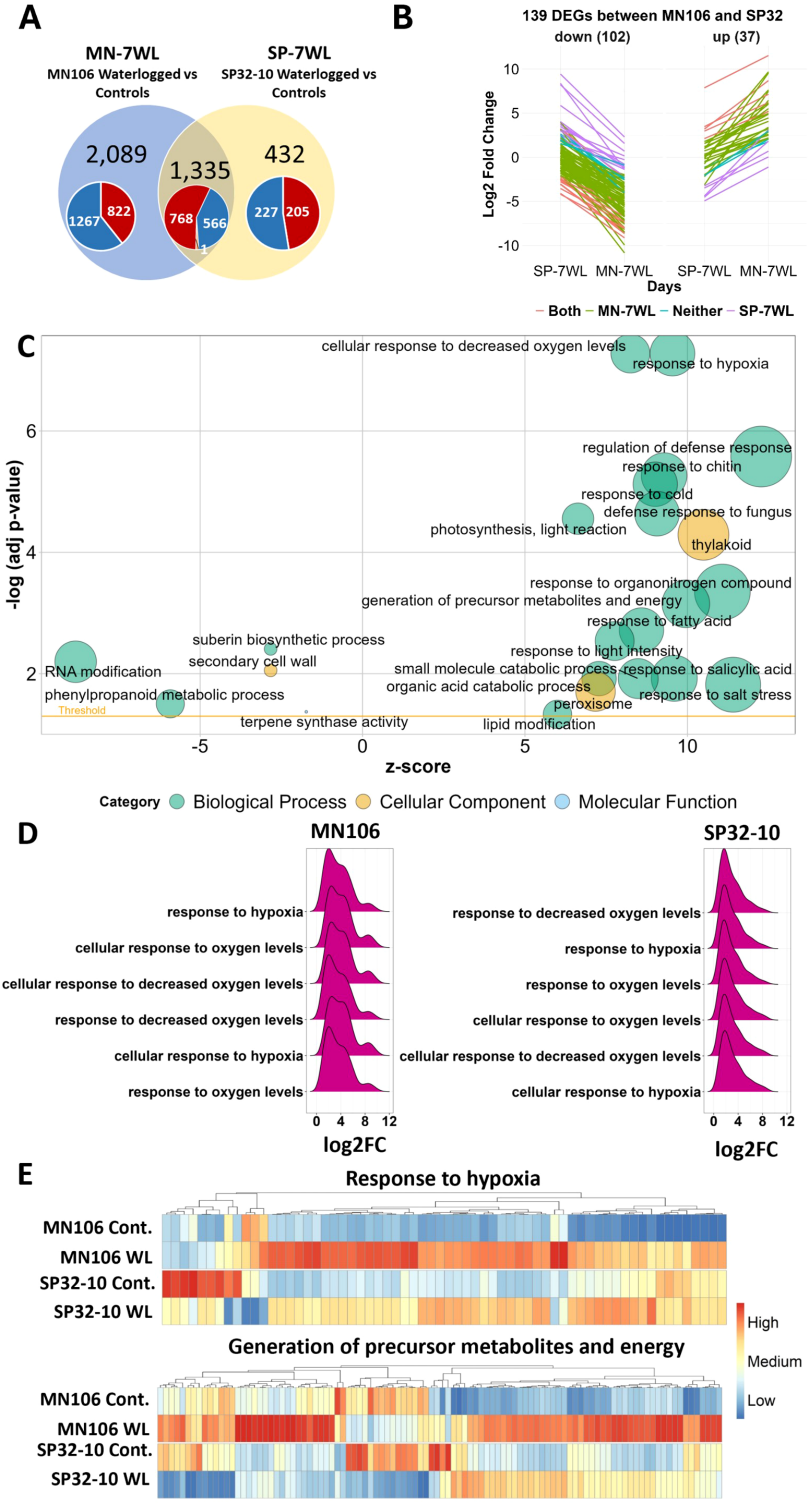
Differentially expressed genes (DEGs) and enrichment results between accessions in waterlogged compared to control roots. **(A)** Venn diagram of overlapping DEGs from MN106 waterlogged *vs* controls (MN-7WL) and SP32-10 waterlogged *vs* controls (SP-7WL). Red, blue, and orange colors in pie chart represent upregulated, downregulated, and contrasting gene expression respectively. **(B)** Visual depiction of the change in log2FC of the 102 down and 37 upregulated DEGs with significant interactions between MN-7WL *vs* SP-7WL. Coral-colored lines indicate genes that were significantly differentially expressed in both MN-7WL and SP-7WL, green and purple lines indicate genes significantly differentially expressed in only MN-7WL and SP-7WL respectively, and blue lines indicate genes that were not significantly differentially expressed in either MN-7WL or SP-7WL (but had significant interactions due to opposite expression patterns). **(C)** Bubble plot of GO terms from GSEA results of the interaction from MN-7WL and SP-7WL. The size of the bubbles is proportional to the number of genes assigned to the term and the z-score provides an estimate if the genes are more likely to be upregulated or downregulated and is calculated by (# of upregulated genes - # of downregulated genes)/√count. **(D)** Ridge plots showing log2FC values of genes falling under hypoxia-related enriched GO terms in the interaction between MN-7WL and SP-7WL. The height of the peak corresponds to the number of genes with the respective log2FC value. **(E)** Heatmap of genes across treatments and accessions falling under the ‘response to hypoxia’ and ‘generation of precursor metabolites and energy’ GO terms from GSEA interaction analysis between MN-7WL and SP-7WL. The input data for the heatmaps were the mean value of normalized count data across biological replicates for each accession and treatment based on the rlog transformation. Heatmap scale was applied by column or gene.

Since there were differences in the morphological and transcriptomic responses of MN106 and SP32-10 in response to 7d waterlogging, we further investigated which DEGs were uniquely differentially expressed in the roots between the accessions. A total of 2,089 genes (61% of total MN-7WL root DEGs) were uniquely differentially expressed in MN-7WL and 432 genes (25% of total SP-7WL root DEGs) were uniquely expressed in SP-7WL (**Figure 4A**). An overrepresentation analysis of the unique DEGs in each accession revealed upregulated DEGs in MN106 (822 total) and SP32-10 (205 total) were involved in lipid and carbon metabolism, phenylpropanoid biosynthesis, and glycolysis KEGG pathways (**Supplementary Table 5**). Therefore, different genes with similar functions were significantly upregulated in these accessions under waterlogging. On the other hand, unique downregulated DEGs in MN106 (1,267 total) were tied to many functions such as secondary metabolite biosynthesis, inorganic ion homeostasis, tropism, root epidermal cell differentiation, and sucrose metabolism (**Supplementary Table 5**). In SP32-10, which had less downregulated unique DEGs than MN106 (227 total), the downregulated DEGs were involved in glycosinolate/glucosinolate metabolism, secondary metabolite biosynthesis, and cell wall biogenesis. Therefore, the unique downregulated DEGs in both accessions were involved in different functions under waterlogging. The unique DEGs suggest MN106 had a more robust response to waterlogging, both in greater number of genes differentially expressed in functional categories affected in both accessions, as well as enrichment of DEGs in functional categories uniquely detected in MN106.

To determine the genes that were significantly differently expressed between accessions under waterlogging, we performed an interaction analysis (treatment x accession) to calculate the difference in gene expression, or the change in log2FC, of all genes in the genome between MN-7WL and SP-7WL. The same cutoffs for determining differentially expressed genes in MN-7WL and SP-7WL were used: genes with a change in log2FC > |1| and an FDR < 0.05 were considered differentially expressed. This revealed 37 DEGs with higher expression and 102 DEGs with lower expression in MN-7WL compared to SP-7WL (**Figure 4B**; **Supplementary File 5**). Out of these 139 DEGs, 95 were also significantly differentially expressed in, and specific to, MN-7WL (71 downregulated and 24 upregulated), whereas 18 were significantly differentially expressed in, and specific to, SP-7WL (13 upregulated and 5 downregulated). The remaining 26 DEGs were either significantly differentially expressed in both MN-7WL and SP-7WL (10 DEGs) or were oppositely expressed between MN-7WL and SP-7WL (16 DEGs). For instance, TAV2_LOCUS6692, a gene encoding a carotenoid isomerase, was upregulated by a log2FC of about 3 in MN-7WL but downregulated by a log2FC of about 2 in SP-7WL, and though this gene was not detected as significantly differentially expressed (log2FC > |1| and FDR < 0.05) in either SP32-10 or MN106 under waterlogging, it had a significant interaction between accessions because the change in log2FC equaled 5 and the FDR was 0.04.

While it is valuable to know the differences in gene expression between the accessions under waterlogging compared to control conditions, it is also important to understand the differences in the baseline, or constitutive expression, between the accessions under control conditions. Therefore, we compared MN106 and SP32-10 control roots and discovered only 120 DEGs (113 with higher expression and 7 with lower expression in MN106 compared to SP32-10). This indicates the accessions had very similar root gene expression under normal watering conditions. A GSEA of the transcript expression between MN106 and SP32-10 control roots identified 24 enriched GO categories, including response to cold, photosystem, response to salt stress, and several categories related to sugar transport (**Supplementary File 2**). The genes within these categories were expressed at lower levels in MN106 compared to SP32-10 control roots. Only two categories had genes with higher expression in MN106 than SP32-10 under control conditions: NAD(P)+ nucleosidase activity and the nucleolus. Of the 139 genes with a significant change in log2FC between MN-7WL and SP-7WL, only 5 were significantly differentially expressed between the accessions under control conditions and all of them were more highly expressed in MN106 under control conditions (indicated in red text in **Supplementary File 5**). Therefore, the majority of the DEGs detected in MN-7WL *vs* SP-7WL waterlogged roots had similar expression under control conditions.

Although only 139 DEGs were detected between MN-7WL and SP-7WL roots, we were able to examine gene enrichment with a GSEA. This revealed that genes with higher expression in MN-7WL compared to SP-7WL were enriched for 39 GO terms, such as response to oxygen levels/hypoxia, regulation of defense response, response to chitin, response to cold, photosynthesis and thylakoid, generation of precursor metabolites and energy, response to fatty acid, peroxisome, and others (**Figure 4C**; **Supplementary File 2**). The functions of the 139 DEGs between MN-7WL and SP-7WL supported these GSEA results. For instance, some of the 37 DEGs with higher expression in MN-7WL *vs* SP-7WL are known to respond to abscisic acid, hypoxia, starvation, and water deprivation (**Supplementary File 5**). Interestingly, *PHOSPHATE STARVATION-INDUCED GENE 2 (ATPS2)* was among the top 10 most significant DEGs in MN-7WL with a log2FC of 8.7, and also had a significant interaction between MN-7WL and SP-7WL with a change in log2FC of 5.5 (**Table 3**). Additionally, upregulated unique genes (no Arabidopsis ortholog) in MN-7WL, based on homology to proteins in other species, are involved in cell wall modification (**Supplementary File 5**).

On the other hand, the GSEA revealed that genes with lower expression in MN-7WL compared to SP-7WL were enriched for 6 GO terms, such as RNA modification, suberin biosynthetic process, secondary cell wall, casparian strip, phenylpropanoid metabolic process, and terpene synthase activity (**Figure 4C**; **Supplementary File 2**). Genes that contributed to the core enrichment of the secondary cell wall and casparian strip GO categories consisted of *ENHANCED SUBERIN 1* (*ESB1*), *LACCASE 1* (*LAC1*), *MYB DOMAIN PROTEIN 39* (*MYB39*), and *CASPARIAN STRIP MEMBRANE DOMAIN PROTEIN 3* (*CASP3*) which were all significantly downregulated in MN-7WL by a log2FC of about 3, except for *CASP3* which was downregulated by a log2FC of about 7 (**Supplementary File 1**). None of these genes were differentially expressed in SP-7WL, and their expression was similar between the accessions under control conditions. Therefore, most of these genes (the latter three) had significant lower expression in MN-7WL compared to SP-7WL (**Supplementary File 5**). Some of the 102 DEGs with lower expression in MN-7WL compared to SP-7WL were also involved in cell wall loosening, lignan biosynthesis, cell growth, casparian strip lignification, iron and sulfur starvation, and root development (**Supplementary File 5**).

Interestingly, we did observe higher expression of genes belonging to several GO categories in MN106 compared to SP32-10 roots under waterlogging. For instance, MN106 waterlogged roots had stronger expression of genes involved in the response to hypoxia than SP32-10, with these genes being expressed twice as high in MN106 compared to SP32-10 (i.e. an average log2FC of about 4 compared to 2, respectively) (**Figures 4D, E**). Some of the genes contributing to the core enrichment of this category were *SENESCENCE ASSOCIATED GENE 14 (SAG14*) and *RESPIRATORY BURST OXIDASE HOMOLOG D (RBOHD)*, which were higher in expression in MN106 waterlogged roots compared to SP32-10. MN106 waterlogged roots also had stronger expression of genes involved in the generation of precursor metabolites and energy (**Figures 4C, E**). Some of the genes contributing to the enrichment of this category consisted of *PYRUVATE KINASE 9*, *FBA3*, *GLYCERALDEHYDE-3-PHOSPHATE DEHYDROGENASE C-2* (*GAPC2*), *FBA6*, and *ENO2*, which were expressed at similar levels under control conditions, but were higher in expression in MN106 under waterlogging (**Supplementary File 1**).

To explore functionally connected genes unique to each accession, we used the MENTOR algorithm (Sullivan et al., 2024) that uses gene expression network topology to assign functionally connected genes into clusters, which are represented in a dendrogram (**Supplementary Figure 6**). This included 913 genes that were differentially expressed in the roots of one genotype but not the other. We used the associated heatmaps to identify large clusters of functionally related genes that were significantly upregulated or downregulated in each genotype to mechanistically interpret genotype-specific responses to waterlogging. Examination of the functional patterns in these clades indicated that many of them were involved in osmotic stress, ion transporters and efflux pumps, hormone regulation and response, and lipid biosynthesis with several connections to suberin. These functional categories corroborate the GO functional categories enriched in the unique downregulated DEGs in MN-7WL (**Supplementary Table 5**). We selected one extended cluster for deeper interpretation based on the unique expression pattern: most genes were downregulated in MN106 under waterlogging stress (outlined with a red box in **Supplementary Figure 6**). In the context of waterlogging stress, the regulation and function of specific pennycress genes exemplified in this functional clade highlights differences in adaptive strategies between MN106 and SP32-10 genotypes, to cope with the challenges posed by excessive water and reduced oxygen availability.

The genes in this clade support a narrative of an intricate balance of water and ion homeostasis, essential for plant survival during waterlogging. For instance, we observed the downregulation of several genes in MN-7WL involved in ion balance and osmotic stability, such as *K+ UPTAKE PERMEASE 6 (KUP6*/TAV2_LOCUS15590/AT1G70300), *POTASSIUM CHANNEL IN ARABIDOPSIS THALIANA 3* (*KAT3/AtKC1*/TAV2_LOCUS22773/AT4G32650), *NOD26-LIKE INTRINSIC PROTEIN 5;1* (*NIP5;1*/TAV2_LOCUS19334/AT4G10380), and *PHO1;H1* (TAV2_LOCUS16940/AT1G68740). One of the few upregulated genes in this functional clade was in MN-7WL and encodes a MATE Efflux Family Protein (TAV2_LOCUS1072/AT1G11670), which could be a response to prevent the accumulation of toxic metabolites in the cells or to maintain hormonal homeostasis under abiotic stress. Furthermore, MN-7WL had several downregulated genes involved in root hydrotropism, meristem growth, and root hair growth, such as a gene encoding the MIZU-KUSSEI-like (MIZ) protein (TAV2_LOCUS5684/AT5G23100), *BARELY ANY MERISTEM 3* (*BAM3*/TAV2_LOCUS24776/AT4G20270), *SKU5 Similar 15* (TAV2_LOCUS24681/AT4G37160), *ROOT MERISTEM GROWTH FACTOR INSENTIVE 4* (*RGI4*/TAV2_LOCUS19419/AT5G56040), *PEROXIDASE 44* (*PRX44*/TAV2_LOCUS24085/AT4G26010), and *GLYCOSYL HYDROLASE 9C1* (*GH9C1*/TAV2_LOCUS3753/AT1G48930). Additionally, there were several downregulated genes involved in suberin and lipid metabolism or transport in MN-7WL, such as genes encoding HXXXD-type acyl-transferase family proteins (TAV2_LOCUS2538/AT1G24430, TAV2_LOCUS5986/AT5G63560, TAV2_LOCUS16047/AT1G78990), *GUARD-CELL-ENRICHED GDSL LIPASE 25* (*GGL25*/TAV2_LOCUS19216/AT5G03610), a gene encoding a GDSL-motif lipase (TAV2_LOCUS13836/AT2G23540), and *GLYCOSYLPHOSPHATIDYLINOSITOL-ANCHORED LIPID PROTEIN TRANSFER 15* (*LTPG15*/TAV2_LOCUS14460/AT2G48130). Lastly, we identified several downregulated genes implicated in hormone responses such as *DIOXYGENASE FOR AUXIN OXIDATION 1* (*DAO1*/TAV2_LOCUS1366/AT1G14130) and *RESPONSIVE-TO-ANTAGONIST 1* (*RAN1*/TAV2_LOCUS6093/AT5G44790), as well as those previously mentioned such as *KUP6*, *PHO1;H1*, *KAT3*, and *NIP5;1.* The genes identified in this functional clade, which were mostly downregulated in MN-7WL and unchanged in SP-7WL, reveal a coordinated response underlining the plant’s ability to acclimate and survive in fluctuating environmental conditions. Our results indicate that MN106 waterlogged roots had stronger regulation of similar stress responses seen in SP32-10 waterlogged roots, but also unique downregulation of genes belonging to categories such as the secondary cell wall, the casparian strip, and water and ion homeostasis.

### Differentially expressed genes following 3 hours of recovery from waterlogging

The sudden removal of a stressful condition can often trigger a stress response in plants, such as the sudden reoxygenation of roots once flood waters recede. To capture the transcriptome response of this environmental switch, we investigated the DEGs between 3h of recovery and controls for each accession (MN-REC and SP-REC) and found 2,398 genes were differentially expressed in MN-REC roots (1,153 upregulated and 1,245 downregulated) and 1,473 genes were differentially expressed in SP-REC roots (791 upregulated and 682 downregulated) compared to controls (**Supplementary File 1**). This is 1,026 DEGs fewer than in MN-WL roots and 294 fewer than SP-WL roots. Furthermore, 2,141 root DEGs in MN106 and 1,168 root DEGs in SP32-10 were shared between 7d of waterlogging and 3h of recovery. This revealed that many genes involved in waterlog stress responses were still activated 3h after the plants had been removed from the water.

We performed an interaction test to identify genes significantly differentially expressed between 3h of recovery *vs*. controls and 7d of waterlogging *vs*. controls. Only 4 upregulated genes related to amino acid biosynthesis in the roots were detected as differentially expressed between MN-REC and MN-7WL (**Supplementary File 1**) and no differential expression between SP-REC and SP-7WL roots was detected, likely because the roots were not fully dried out and the recovery tissues were very similar to the waterlogged tissues (**Supplementary Figure 7**).

Although very few DEGs were detected between MN-REC *vs* MN-7WL and SP-REC *vs* SP-7WL, we conducted a GSEA to examine changes in expression of the entire transcriptome landscape between recovery and waterlogging. These results indicated that in the few hours after the plants were removed from the waterlogged environment, some initial stress responses were lowered, such as the response to hypoxia, and other cellular and metabolic processes were increased, such as secondary metabolite biosynthesis (**Supplementary Files 2**, **3**). Therefore, while many of the same genes were differentially expressed at 3h of recovery compared to 7d of waterlogging, these genes had slight changes (less than a log2FC = |1|) in expression levels.

### N-degron pathway gene expression

**Figure 5:**
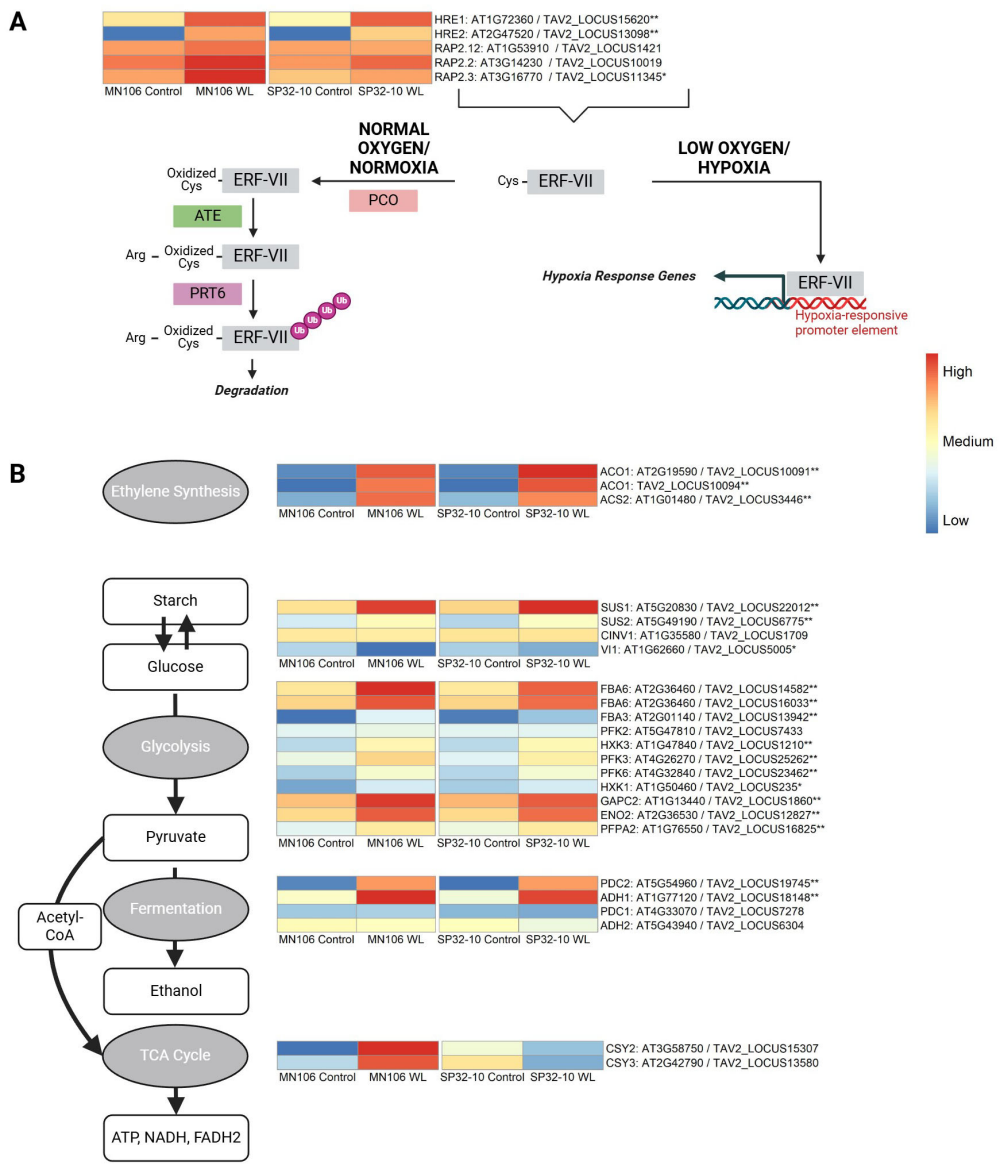
Heatmap of genes involved in the N-degron pathway and core anaerobic processes. **(A)** Under normal oxygen conditions (normoxia), the ERF-VII transcription factor family proteins are susceptible to the N-degron pathway where plant cysteine oxidases (PCO) oxidate the N-terminus cysteine residue, followed by arginylation by (ATE) and are tagged for ubiquitination by proteolysis 6 (PRT6) for degradation. Under hypoxia, the ERF-VII proteins remain stable and bind to hypoxia response promoter elements of genes involved in anaerobic responses such as **(B)** starch and glucose biosynthesis/degradation, glycolysis, fermentation, and the TCA cycle. The input data for the heatmaps were the mean value of normalized count data across biological replicates for each accession and treatment based on the rlog transformation. The heatmap scale/colors were assigned based on the mean value of expression across all replicates for each condition and accession for all genes within each individual heatmap. A single asterisk next to the gene name indicates differential expression in waterlogged compared to control roots in MN106, and double asterisks indicate differential expression in both MN106 and SP32-10 waterlogged compared to control roots. Figure adapted from Combs-Giroir and Gschwend, 2024.

The Group VII Ethylene Response Factors (ERF-VIIs) are stabilized under hypoxia via the N-degron pathway and play a role in hypoxic stress responses (**Figure 5A**) (Gibbs et al., 2011, 2015). This family consists of five members in Arabidopsis (*HYPOXIA RESPONSIVE 1/HRE1*, *HRE2*, *RELATED TO APETALA/RAP2.12*, *RAP2.2*, and *RAP2.3*) (Giuntoli and Perata, 2018). We wanted to determine if the genes encoding ERF-VIIs, and the genes regulated by ERF-VIIs, were differentially expressed in pennycress roots after waterlogging, and if there were differences in expression between the two accessions. The genes encoding HRE1 (TAV2_LOCUS15620) and HRE2 (TAV2_LOCUS13098) were significantly upregulated in both MN-7WL and SP-7WL roots by a log2FC of about 3 in MN-7WL and 4 in SP-7WL for *HRE1* and about 9 in MN-7WL and 7 in SP-7WL for *HRE2* (**Figure 5A**). Interestingly, the log2FC of *HRE2* was twice that of *HRE1*. *RAP2.3* (TAV2_LOCUS11345) was only differentially expressed (upregulated) in MN-7WL roots, but not in SP-7WL. Lastly, *RAP2.12* (TAV2_LOCUS1421) and *RAP2.2* (TAV2_LOCUS10019) showed no significant differential expression in the waterlogged roots in either accession. Furthermore, none of the *ERF-VII* genes were significantly differentially expressed between the two accessions (MN-7WL *vs* SP-7WL) under waterlogging.

Under low oxygen, ERF-VII transcription factors bind to hypoxia response promoter elements (HRPE) of core hypoxia response genes (Licausi et al., 2010; Gasch et al., 2016), such as those involved in sugar metabolism (*SUS*), glycolysis, and fermentation (*ADH, PDC*). In MN-7WL and SP-7WL roots, *PDC2* and *ADH1* were in the top 10 most significant differentially expressed upregulated genes with a log2FC of about 8.5 and 6, respectively (**Table 3**). Genes involved in the glycolysis pathway were enriched in both MN-7WL and SP-7WL roots, with significantly upregulated genes such as *FBA6*, *PHOSPHOFRUCTOKINASE 3* and 6/*PFK3* and *PFK6*, and *HXK3*, with log2FC’s of about 4-5 (**Figure 5B**; **Supplementary File 4**). *SUS1-like* (TAV2_LOCUS22012) was upregulated about 4-log2FC in MN-7WL, and SP-7WL roots (**Figure 5B**). *SUS2-like* (TAV2_LOCUS6775) was also upregulated about 3-log2FC in MN-7WL and SP-7WL roots. Lastly, expression levels of a cytosolic invertase (*CINV1/*AT1G35580/TAV2_LOCUS1709) were slightly downregulated in waterlogged roots in both accessions, suggesting the favoring of sucrose synthase over invertase under waterlogging (**Supplementary File 1**). These results reveal that some *ERF-VII* genes (*HRE1*, *HRE2*, and *RAP2.3*) are differentially expressed in pennycress roots under waterlogging, and the genes activated by these transcription factors are very highly expressed (*PDC*, *ADH*, *SUS*), however, the expression levels of these genes are very similar between accessions.

## Discussion

### Waterlogging caused reduced growth and impacted seed yield and oil quality

Pennycress is being developed as a cash crop, meaning that the seed is harvested and crushed for oil used for biofuel production. Therefore, it is valuable to understand how extreme weather events like flooding could impact pennycress seed yield and oil quality and if pennycress accessions respond differently. At maturity, we observed a reduction in biomass and yield in waterlogged plants, suggesting a halt in pennycress growth as a result of the severe energy crisis invoked by waterlogging. This was an expected response, as many waterlogging studies in *Brassicaceae* species have also reported reduced biomass and/or seed yield under waterlogging, such as with *Brassica napus* (Zou et al., 2014; Xu et al., 2015; Ploschuk et al., 2018, 2021; Wollmer et al., 2018; Liu et al., 2020; Liu and Zwiazek, 2022), *Brassica rapa* (Daugherty and Musgrave, 1994; Daugherty et al., 1994), *Brassica oleracea* (Issarakraisila et al., 2007; Casierra-Posada and Peña-Olmos, 2022), and *Arabidopsis thaliana* (Pigliucci and Kolodynska, 2002). The reduction in plant growth and development under abiotic stress, such as flooding, could be attributed to multiple factors, such as reduced ATP synthesis and photosynthetic rates (Bailey-Serres and Voesenek, 2008; Xu et al., 2019), nutrient deficiency (Steffens et al., 2005), or a trade-off with mounting a defense or acclimation response (Bechtold and Field, 2018).

**Figure 6:**
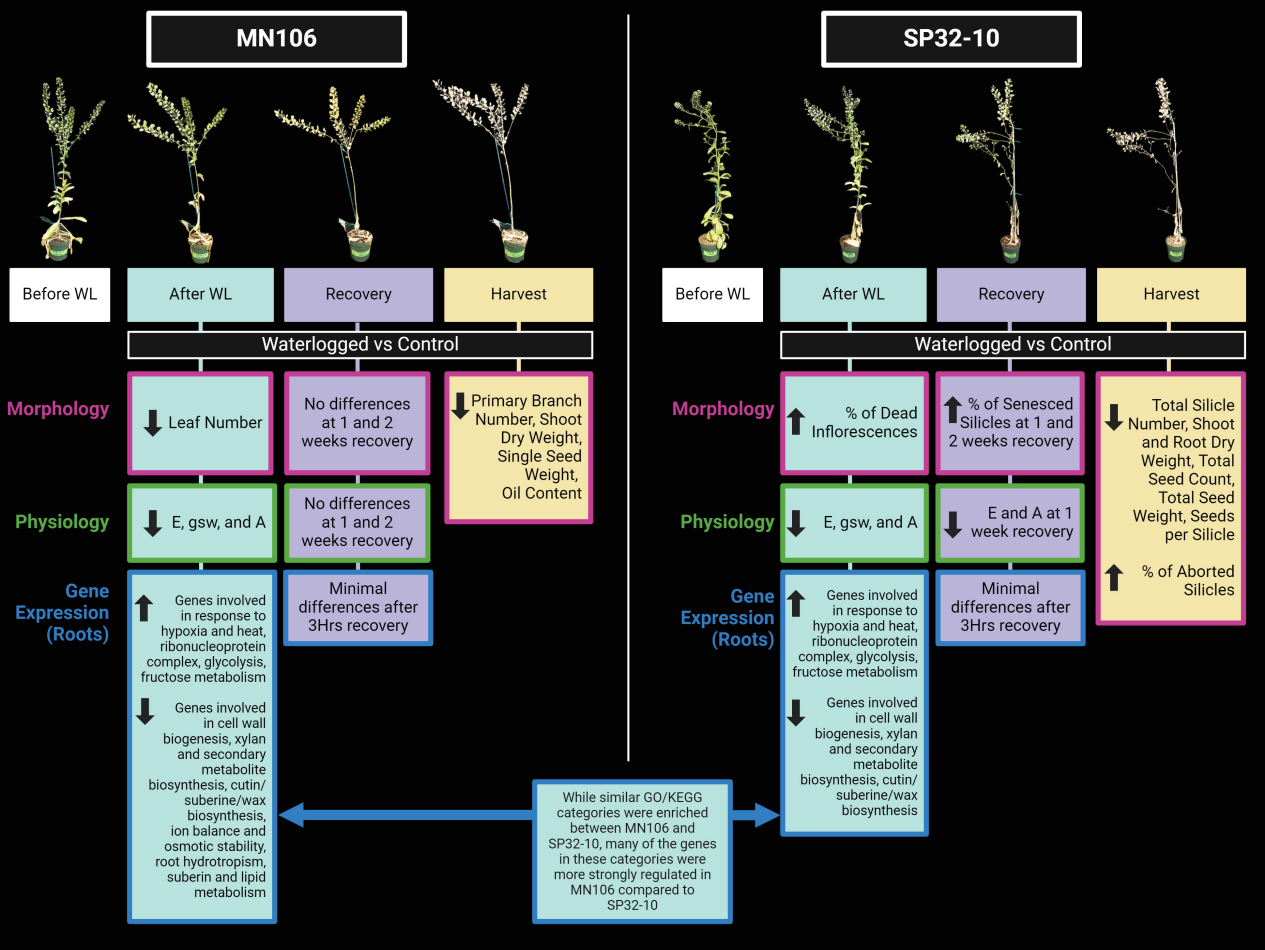
Model summarizing the significant changes in morphology, physiology, and root gene expression between waterlogged and control plants in each accession after waterlogging, during recovery, and at harvest. The top panel is a display of a single waterlogged replicate immediately before waterlogging (white box), after 7d of waterlogging (blue box), after 2 weeks of recovery (purple box), and at the time of harvest/maturity (yellow box). Underneath the latter three timepoints are boxed summaries of which morphological (pink outline) or physiological (green outline) traits were significantly reduced (down arrow) or increased (up arrow) in waterlogged compared to control plants. Boxes with a blue outline summarize some of the key findings for root gene expression after 7d of waterlogging and after 3hr of recovery, where the up arrow indicates upregulation, and the down arrow indicates downregulation. WL, waterlogging; E, transpiration rate; gsw, stomatal conductance; A, CO2 assimilation.

Additionally, more morphological traits, including seed yield traits and root dry weight, were negatively impacted by waterlogging in SP32-10 than MN106 (**Figure 6**), indicating variation in pennycress accessions fitness under waterlogging stress. Similar to pennycress, differences in seed yield outcomes in *B. napus* after reproductive-stage waterlogging may also be attributed to differences in genotypes (Zhou and Lin, 1995; Ploschuk et al., 2018, 2020, 2021, 2023). While biomass was not significantly reduced in either accession directly after waterlogging, root dry weight was significantly reduced in SP32-10 at the time of harvest. It is possible that MN106 halted growth to conserve energy until the water receded, similar to *Rorippa sylvestris*, lowland rice cultivars, and *Rumex acetosa* under full submergence (Fukao et al., 2006; Akman et al., 2012; van Veen et al., 2013), and with some evidence in *B. napus* and *A. thaliana*, as well (Lee et al., 2011; Wittig et al., 2021). Our results suggest pennycress yield can be negatively impacted by waterlogging stress and that different accessions can be impacted differently by waterlogging.

Our transcriptome data revealed several differences between the accessions under waterlogging that might contribute to these differences in biomass and yield (**Figure 6**). Genes within many GO categories and pathways related to cell wall biogenesis, plant epidermis development, and the cell cycle were significantly downregulated in waterlogged plants, with lower expression in MN-7WL than SP-7WL roots. A similar observation was reported in a *B. napus* study that compared gene expression between seedlings of a tolerant and sensitive variety under waterlogging (Zou et al., 2015); The DEGs that overlapped between the two varieties under waterlogging had similar expression patterns, except most of the overlapping downregulated genes, such as those involved in carbohydrate metabolism, were more strongly downregulated in the tolerant variety, possibly to limit energy consumption or growth. Additionally, waterlogging at the germination stage in *B. napus* also showed downregulation of “starch and sucrose metabolism” and “photosynthesis” related genes that can explain the observed reduced development of the hypocotyl, shoot, and roots, as well as differential expression of several genes that might play a role in cell differentiation and division in the root apical meristem, such as *INDOLE-3-ACETIC ACID INDUCIBLE 17/IAA17* and *CRINKLY4*/*ACR4* (Guo et al., 2020). In MN-7WL roots, genes enriched for root epidermal cell differentiation were downregulated and an *IAA17*-like gene (*TAV2_LOCUS6916*) and *ACR4*-like gene (*TAV2_LOCUS15261*) were significantly differentially expressed (down and upregulated respectively), suggesting the inhibition of root development. This was not observed in SP32-10 waterlogged roots. Other downregulated genes were also linked to root growth and development, including a *MIZ* gene, *BAM3*, *SKS15*, *PRX44*, and *GH9C1*. In Arabidopsis, MIZ1, a representative of the MIZU-KUSSEI-like (MIZ) proteins, is essential for root hydrotropism, or the directional growth of roots toward moisture gradients (Moriwaki et al., 2011). Similarly, *BAM3* is essential in protophloem differentiation and root meristem growth in Arabidopsis (Depuydt et al., 2013), and *SKU5*, a homolog of *SKS15*, affects directional growth processes (Sedbrook et al., 2002). The *PRX44* gene, which encodes a class-III peroxidase, has been proposed to be involved in the polar expansion of root hair cells by controlling extensin-mediated cell wall properties (Marzol et al., 2022), and the *GH9C1* gene has also been proposed to be involved in root hair growth through cell wall weakening (Campillo et al., 2012). Furthermore, *DAO1* and *RAN1* were downregulated in our dataset. In Arabidopsis, *DAO1* is vital for maintaining auxin at optimal levels and *RAN1* is essential for ethylene signaling, suggesting these genes likely play an important role in hormone signaling in pennycress, as well (Woeste and Kieber, 2000; Mellor et al., 2016). In conclusion, many genes involved in root cell growth were more downregulated in MN106 waterlogged roots than SP32-10, possibly as a method to conserve energy which may have contributed to the ability of MN106 to recover more quickly and resume growth after the waterlogging period.

A primary concern of flooding events is the impact on pennycress seed oil content. Total seed oil content is of greatest significance for pennycress, since the oil is directly used downstream for biofuel production. In our study, pennycress seed oil content was not significantly reduced after waterlogging in our greenhouse experiment, but there was a significant reduction in total oil content for MN106 in our growth chamber experiment, suggesting waterlogging could negatively affect oil content for some pennycress accessions under certain conditions. A waterlogging study with *B. napus* at early flowering stages in the field reported negative impacts on oil quality, but no significant changes in oil content (Xu et al., 2015). Changes in some pennycress oil constituents after waterlogging were detected in our study but were the opposite of what was observed in *B. napus*. Xu et al. (2015) reported increased erucic acid and decreased linoleic acid in *B. napus* after reproductive-stage waterlogging, whereas MN106 waterlogged seeds had decreased erucic acid and increased linoleic acid, however, these discrepancies could be attributed to differences in environmental conditions. The consequence of differences in oil properties depends on the end-use of the oil. For instance, the high erucic content of wild pennycress seed makes the oil suitable for aviation fuel and biodiesel production but is unsuitable for human consumption (Moser et al., 2009; Isbell et al., 2015). With abolished erucic acid in mutant pennycress varieties (*fae1*), the oil could have potential food applications (McGinn et al., 2019; Buck et al., 2022). Since fatty acid profiles play an important role in the end-use of the oil, it is crucial to understand how abiotic stress like waterlogging might impact the seed oil profiles. While there have been reports on changes to seed oil fatty acid composition in *B. napus* following reproductive-stage waterlogging, no studies have analyzed gene expression in seed tissues during or after waterlogging. In our RNA-seq dataset, the GSEA detected that genes with increased expression in MN-7WL silicles were enriched in functions related to fatty acid and lipid responses, and genes with decreased expression were enriched for fatty acid biosynthesis such as genes involved in VLCFA synthesis. This could be the reason for decreased levels of VLCFAs in the seed oil of MN106 waterlogged plants, such as eicosenoic, behenic, and erucic acid, which was not observed in SP32-10.

### Waterlogging caused higher expression of genes involved in energy production in MN106

Under flooding and/or hypoxia stress in plants, mitochondrial respiration is severely compromised by a lack of oxygen, causing an increase in glycolysis, ethanol fermentation, and sugar metabolism (Sairam et al., 2008). The accumulation of pyruvate from glycolysis is converted to acetaldehyde by PDC, and ADH then reduces acetaldehyde to ethanol to facilitate the regeneration of NAD+ from NADH. While this helps to maintain energy production, it is an inefficient method because only 2 ATP are generated compared to approximately 36 ATP produced under aerobic respiration. In the roots of both accessions under waterlogging, *PDC2* and *ADH1* were very highly differentially expressed, and genes involved in glycolysis were upregulated. Upregulation of these genes, or increased enzyme activity, has also been observed in *B. napus* and *B. rapa* roots under waterlogging (Daugherty and Musgrave, 1994; Daugherty et al., 1994; Balakhnina et al., 2012; Xu et al., 2016), and similar to pennycress, no differences in *PDC* or *ADH* transcript levels were detected in *B. napus* cultivars with varying waterlogging tolerance (Xu et al., 2016). However, some genes involved in the generation of precursor metabolites and energy had higher expression in MN106 compared to SP32-10 waterlogged roots, which were mostly glycolysis genes such as *FBA6*, *GAPC2*, and *ENO2.* We also identified cases of tradeoffs to increase energy production. For example, the downregulation of genes involved in lignin and suberin biosynthesis in waterlogged MN106 roots was an unexpected result since these components can serve beneficial roles under flooding, such as formation of radial oxygen-loss (ROL) barriers to prevent oxygen leakage and entry of soil-derived gases and phytotoxic substances (Ejiri et al., 2021; Jiménez et al., 2021). However, the downregulation of these genes might be a mechanism for conserving growth and energy in response to hypoxia. Soluble sugars, specifically glucose, are in demand under waterlogging, which potentially sends more phosphoenolpyruvate to glycolysis rather than the shikimate pathway where the precursor for lignin, phenylalanine, is produced (Vanholme et al., 2012). Therefore, the downregulation of lignin genes in MN106 might instead be a response to the increasing need for phosphoenolpyruvate to support glycolysis under decreased oxygen in the roots. These results showcase the ability of MN106 to strongly upregulate glycolysis in waterlogged roots to sustain energy production.

Furthermore, genes encoding pyruvate kinase (PK)/pyruvate phosphate dikinase (PPDK) cycle proteins, which synthesizes inorganic pyrophosphate (PPi), were significantly upregulated in waterlogged roots of both accessions, but had higher expression in MN106 than SP32-10. This includes a *pyrophosphate-(PPi) dependent pyrophosphate-fructose-6-phosphate-phosphotransferase* (PFPA2: TAV2_LOCUS16825/AT1G76550) with a change in log2FC of 0.6, pyruvate orthophosphate dikinase (PPDK: TAV2_LOCUS23902/AT4G15530) with a change in log2FC of 0.9, and a proton pumping membrane-bound pyrophosphatase (mPPase: TAV2_LOCUS15230/AT1G15690) with a change in log2FC of 1.7. This could indicate PPi production for glycolysis is a mechanism for improved waterlogging tolerance. For instance, anoxia-tolerant species, such as rice, have been shown to use PPi as high-energy donor molecules under ATP deficiency, where PPi can be synthesized from the PK/PPDK cycle or in the reverse reaction of membrane-bound H^+^-pyrophosphatase (Huang et al., 2008; Igamberdiev and Kleczkowski, 2021). Oppositely, the absence of PPi-dependent glycolysis has been observed in *Arabidopsis thaliana*, an anoxia-intolerant species (Loreti et al., 2005; Igamberdiev and Kleczkowski, 2021). Interestingly, gene clusters differentially regulated between two flooding-tolerant *Rorippa* species and *A. thaliana* exposed genes associated with this pathway, indicating this could be a shared flooding tolerance mechanism to support energy metabolism within the *Brassicaceae* family (Sasidharan et al., 2013). Consequently (PPi)-dependent alternative pathways of phosphorylation are worth future exploration for improved waterlogging tolerance in pennycress.

### Waterlogging caused upregulation of ERF-VII transcription factors and sucrose synthase genes in both accessions

The ERF-VII transcription factor family has been reported to play a role in hypoxia. For instance, RAP2.2, RAP2.12, and RAP2.3 are involved in the activation of hypoxia-responsive genes, such as those involved in energy production under oxygen deficit (Gasch et al., 2016). Among these three genes, we only saw significant upregulation of *RAP2.3* in MN106 waterlogged roots, however, the ERF-VII genes are mostly regulated post-translationally through targeted proteolysis via oxygen sensing (Gibbs et al., 2011, 2015; Licausi et al., 2011). Although HRE1 and HRE2 only play a minor role in the activation of hypoxia-response genes in Arabidopsis, they might contribute to hypoxia tolerance through other mechanisms, such as ROS homeostasis (Park et al., 2011; Yao et al., 2017). These genes were significantly upregulated in waterlogged roots in both accessions, with *HRE2* expression doubled compared to *HRE1* and with higher expression in MN106 than SP32-10 (1.7 log2FC difference). Previous research has shown that *HRE1* requires protein synthesis of transcription factors to be induced, whereas *HRE2* is likely to be constitutively expressed, but its mRNA is unstable under normoxia (Licausi et al., 2010). The same study also proposed that *HRE1* might upregulate *HRE2*. This could be a potential reason for why *HRE2* is more strongly upregulated under waterlogging in pennycress. Strong induction of *HRE2* was also found in dark-submerged *Rorippa* roots (van Veen et al., 2014) and hypoxic Arabidopsis roots (Licausi et al., 2010), possibly indicating a conserved response across *Brassicaceae* when roots are exposed to hypoxic environments.

Both pennycress accessions also had increased expression of *SUS1* and *SUS2* in waterlogged roots. Sucrose synthase, which catalyzes the reversible cleavage of sucrose into fructose and UDP or ADP-glucose, is suggested to play a beneficial role in alleviating anaerobic stress by providing an energetically efficient method of supplying UDP-glucose for biological processes (Stein and Granot, 2019). Elevated sucrose synthase transcript and protein abundance were also detected in response to flooding and hypoxia in several other species, including wheat (*Triticum aestivum*) (Albrecht and Mustroph, 2003), potato (*Solanum tuberosum*) (Biemelt et al., 1999), *Arabidopsis thaliana* (Loreti et al., 2005; Bieniawska et al., 2007), rice (*Oryza sativa*) (Locke et al., 2018), cucumber (*Cucumis sativus*) (Kęska et al., 2021), and zombi pea (*Vigna vexillate*) (Butsayawarapat et al., 2019). Sucrose synthase mutants have also been observed to perform poorly under flooding or hypoxic growth conditions (Bieniawska et al., 2007; Yao et al., 2020), further supporting its important role in energy production under oxygen deficiency.

### Genes involved in water and ion homeostasis were downregulated in waterlogged MN106 roots

**Figure 7:**
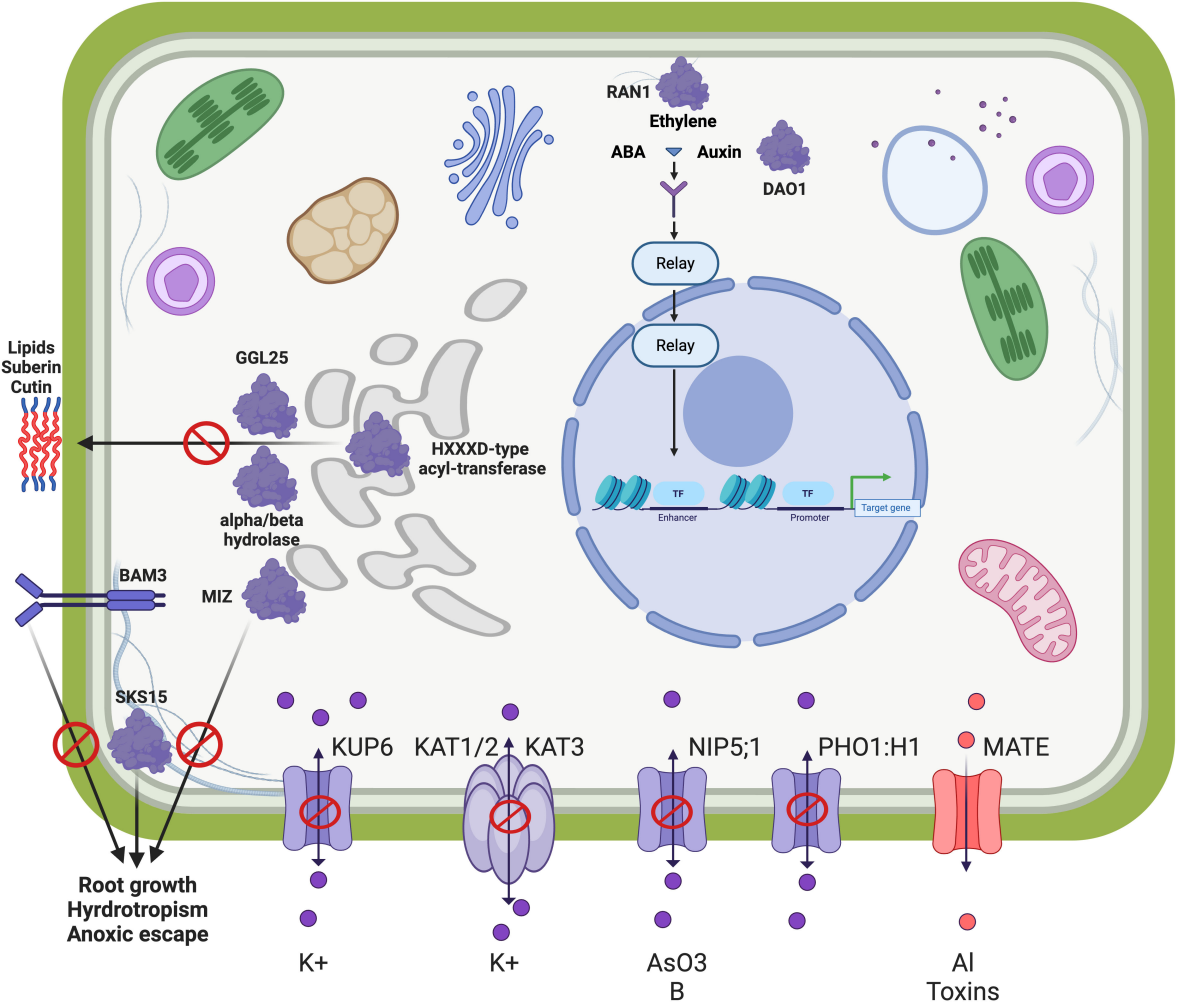
Conceptual model of a functional clade of genes differentially expressed between MN106 and SP32-10. These genes do not represent the major changes under waterlogging between the two genotypes, but rather genes that were differentially expressed and clustered into a functional clade where most genes were downregulated in MN106. The downregulation of inward transport associated with *KUP6*, *KAT3*, *NIP5:1*, and *PHO1;H1* and upregulation of eflux pump MATE helps to maintain osmotic balance and cell health. The downregulation of *HXXXD-type acyl transferases*, *alpha/beta hydrolase*, and *GGL24* decreases the production of suberin/cutin, since water conservation is not required during waterlogging. Similarly, *MIZ*, *SKS15*, and *BAM3* are downregulated as hydrotropism is not required during waterlogging. Hormones play an active role in the transcriptional regulation of many of these genes, including *RAN1*, which regulates the ethylene response pathways and *DAO1* that affects auxin levels.

Another unique pattern between MN106 and SP32-10 under waterlogging was the significant downregulation of a set of genes involved in water and ion homeostasis in MN106 (**Figure 7**). Genes involved in lignin and suberin biosynthesis were found to be downregulated in waterlogged MN106 roots. Casparian strips are apoplastic barriers composed of lignin and suberin in a ring-like structure around endodermal cells and regulate what enters the vascular tissues (Naseer et al., 2012). Genes with lower expression in MN106 compared to SP32-10 were enriched for casparian strip and secondary cell wall categories. Genes encoding peroxidases and a putative casparian strip membrane protein, which are required for casparian strip lignification (Rojas-Murcia et al., 2020), had significant lower expression in MN106 waterlogged roots compared to SP32-10, indicating that waterlogged MN106 roots might have had disrupted, or discontinuous, casparian strips and reduced lignin deposits in root cells which could affect nutrient homeostasis or apoplastic transport. Reductions in lignin content have been observed in wheat (Nguyen et al., 2016) and soybean (Komatsu et al., 2010) under waterlogging or submergence. Suberin is a hydrophobic biopolymer composed of fatty acids that forms a protective barrier, playing a role in water, solute, and gas regulation. Genes encoding HXXXD-type acyl-transferase family proteins, which are involved in the biosynthesis of lipid polymers that are components of the cuticle and suberized cell walls (Kosma et al., 2012), were downregulated in MN106 roots during waterlogging. Also, GGL25 is part of the larger GDSL-type esterase/lipase gene family and is involved in lipid metabolism (Lai et al., 2017). GGL25 and a GDSL-type esterase/lipase gene were more downregulated in MN106 than SP32-10 roots, indicating a role in osmotic stress response (Ding et al., 2019). The downregulation of these genes suggest their involvement in lipid metabolism and modification of extracellular structures, such as suberin in pennycress roots, under waterlogging, and could be part of a broader metabolic adjustment to prioritize energy conservation and reallocation of resources to more critical survival processes, such as anaerobic respiration (Molina and Kosma, 2015).

Another important component of the adaptive response to waterlogging is the regulation of ion homeostasis. For example, potassium is a key element in maintaining cell turgor and osmotic balance (Osakabe et al., 2014), so the downregulation of the *KUP6* potassium transporter in MN106 is likely a significant player in MN106 potassium homeostasis. Complementing this is the downregulation of the *KAT3* gene, also known as *AtKC1*, which, along with KAT1, KAT2, and AKT2, is part of the Shaker family of voltage-gated potassium channels in *Arabidopsis thaliana*. KAT1, an inward-rectifying channel, is pivotal for the inward transport of potassium ions (K+) into the cells, resulting in water influx through osmosis, causing cells to swell (Schachtman et al., 1992; Uozumi et al., 1995; Saponaro et al., 2017; Locascio et al., 2019) and KAT3 can form heteromeric channels with these inward-rectifying channels, altering their conductance and shifting their activation potential (Jeanguenin et al., 2011). *NIP5;1*, a member of the Nodulin-26-like Intrinsic Protein (NIP) family, was also downregulated in MN106 waterlogged roots. In Arabidopsis, NIP5;1 acts as an aquaporin and facilitates the transport of boric acid and arsenite, in addition to water, across cellular membranes, affecting root hydraulic conductance and nutrient uptake (Takano et al., 2006; Martinez-Ballesta et al., 2008; Mitani-Ueno et al., 2011). Additionally, *PHO1;H1*, a gene that plays a crucial role in inorganic phosphate (Pi) transport and homeostasis in Arabidopsis, was downregulated in MN106 waterlogged roots. PHO1;H1 contributes to Pi loading into the xylem and is regulated by Pi deficiency (Stefanovic et al., 2007). Collectively, *KUP6*, *KAT3*, *PHO1;H1*, and *NIP5;1* likely contribute to ion balance and osmotic stability, with their downregulation suggesting a critical role in the plant’s adaptation to waterlogged soils (Philippar et al., 2004).

A gene encoding a MATE Efflux Family Protein was upregulated in MN106 under waterlogging. MATE proteins transport a wide range of substrates, including organic compounds, plant hormones, and secondary metabolites, out of cells and play a critical role in various plant processes and stress responses. In the context of waterlogging stress, the upregulation of MATE efflux proteins could be attributed to several factors, including detoxification and transport of plant hormones. During waterlogging, plants may experience an accumulation of toxic metabolites due to reduced oxygen availability and altered metabolic processes. The upregulation of a MATE under such conditions could be a protective mechanism to remove harmful compounds from the cells, thereby aiding in stress tolerance and survival (Liu et al., 2009; Duan et al., 2013). Waterlogging stress also alters the hormonal balance in plants, impacting growth and development. The upregulation of MATE efflux proteins may also contribute to hormonal homeostasis under stress conditions (Upadhyay et al., 2020). The upregulation of MATE efflux family proteins during waterlogging stress is likely an adaptive response to manage the internal cellular environment and help the plant mitigate the adverse effects of waterlogging to maintain its physiological functions. **Figure 7** shows a conceptual model of how the products of these differentially expressed genes may be involved in a waterlogging stress response.

## Future perspectives

In conclusion, waterlogging in pennycress during the reproductive stage can impair growth, development, and yield, and provoke the reconfiguration of metabolic processes and activation of key stress responses. However, a remaining question is the performance of pennycress under waterlogging in field environments, particularly in response to heavy spring precipitation, but also to repeated flood events throughout the growing season. Additionally, pennycress fields are susceptible to standing water following snow melt during the winter, leading to partial or full submergence of rosettes. The impact of flooding stress on pennycress phenology, growth, yield, and oil quality in field environments at rosette and reproductive stages should be further evaluated. Moreover, the addition of metabolomic analyses of waterlogged pennycress will elucidate and/or provide additional insight into the results reported here, particularly in relation to the utilization of carbohydrate reserves for survival. Furthermore functional validation of candidate genes involved in waterlogging tolerance, including *SUS1*, *HRE2*, *ATPS2*, *RBOHD*, and *SAG14*, will equip breeders with new knowledge for the development of resilient pennycress lines, with strong potential for translation to other *Brassicaceae* crops (Chopra et al., 2018; McGinn et al., 2019). As a cash crop in the early stages of commercialization, the quick development and incorporation of climate-resilient pennycress will result in a more robust and successful crop.

## Data Availability

The datasets presented in this study can be found in online repositories. The names of the repository/repositories and accession number(s) can be found in the Supplementary Material as well as below: https://www.ncbi.nlm.nih.gov/sra/PRJNA1135492.

## Supplementary material

Supplementary files can be accessed at https://github.com/combsgiroir/Supplemental_Data_Waterlogging_Transcriptome_Paper
