# Supplementary material for: Morpho-physiological and transcriptomic responses of field pennycress to waterlogging
Source: Front Plant Sci. 2024 Dec 18;15:1478507. doi: 10.3389/fpls.2024.1478507 (PMC11688638; doi:10.3389/fpls.2024.1478507)
Supplement: Supplementary file 1 [file DataSheet1.pdf]

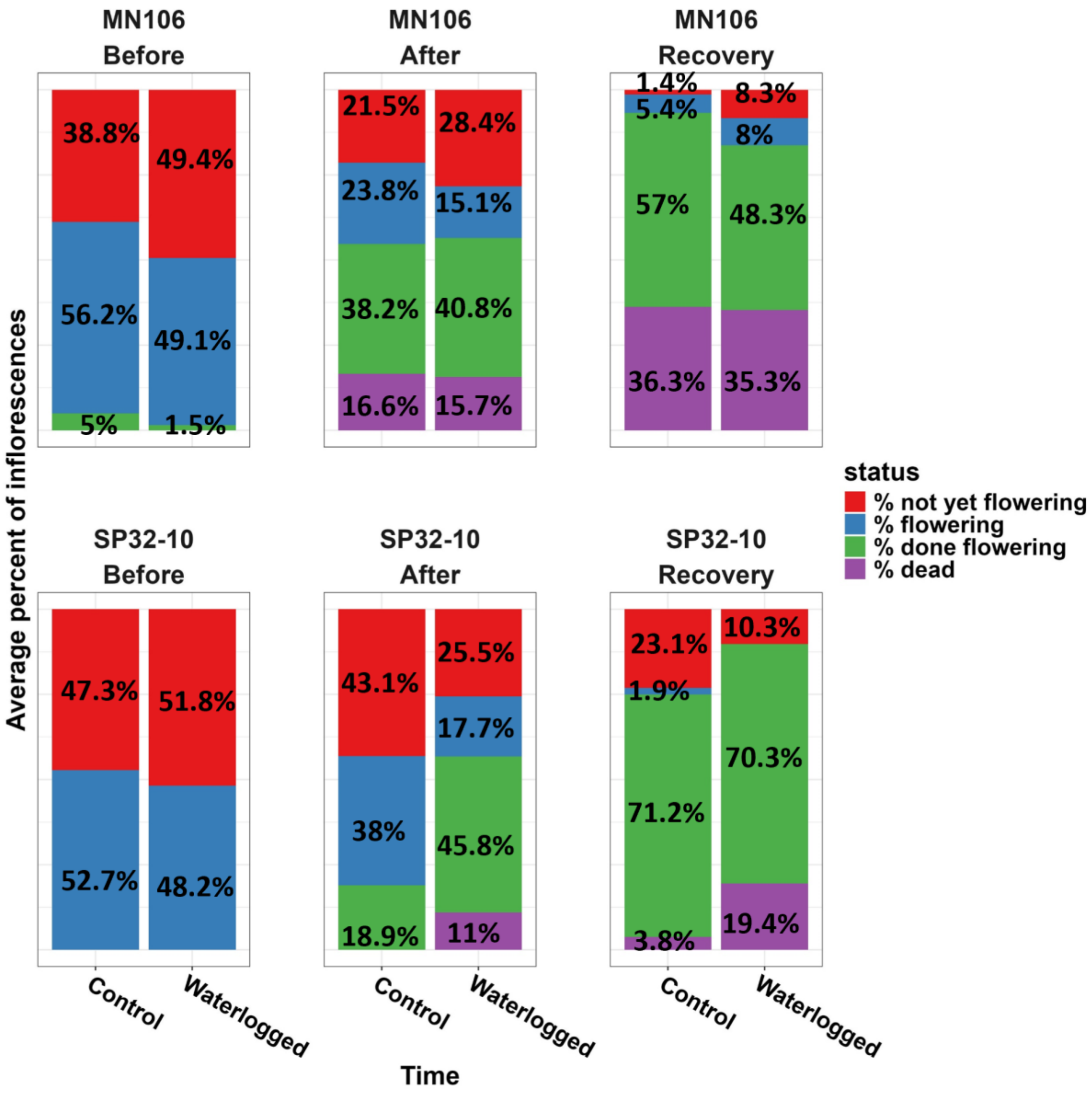

Supplementary Figure 1. Stacked bar plots representing the mean status of inflorescences immediately before waterlogging, immediately after the 7d waterlogging treatments, and after 1 week of recovery from the 7d waterlogging treatments of MN106 and SP32-10 in the growth chamber experiment. Percentages were based on the status of inflorescences divided by the total number of inflorescences per plant. Sample size = 6.

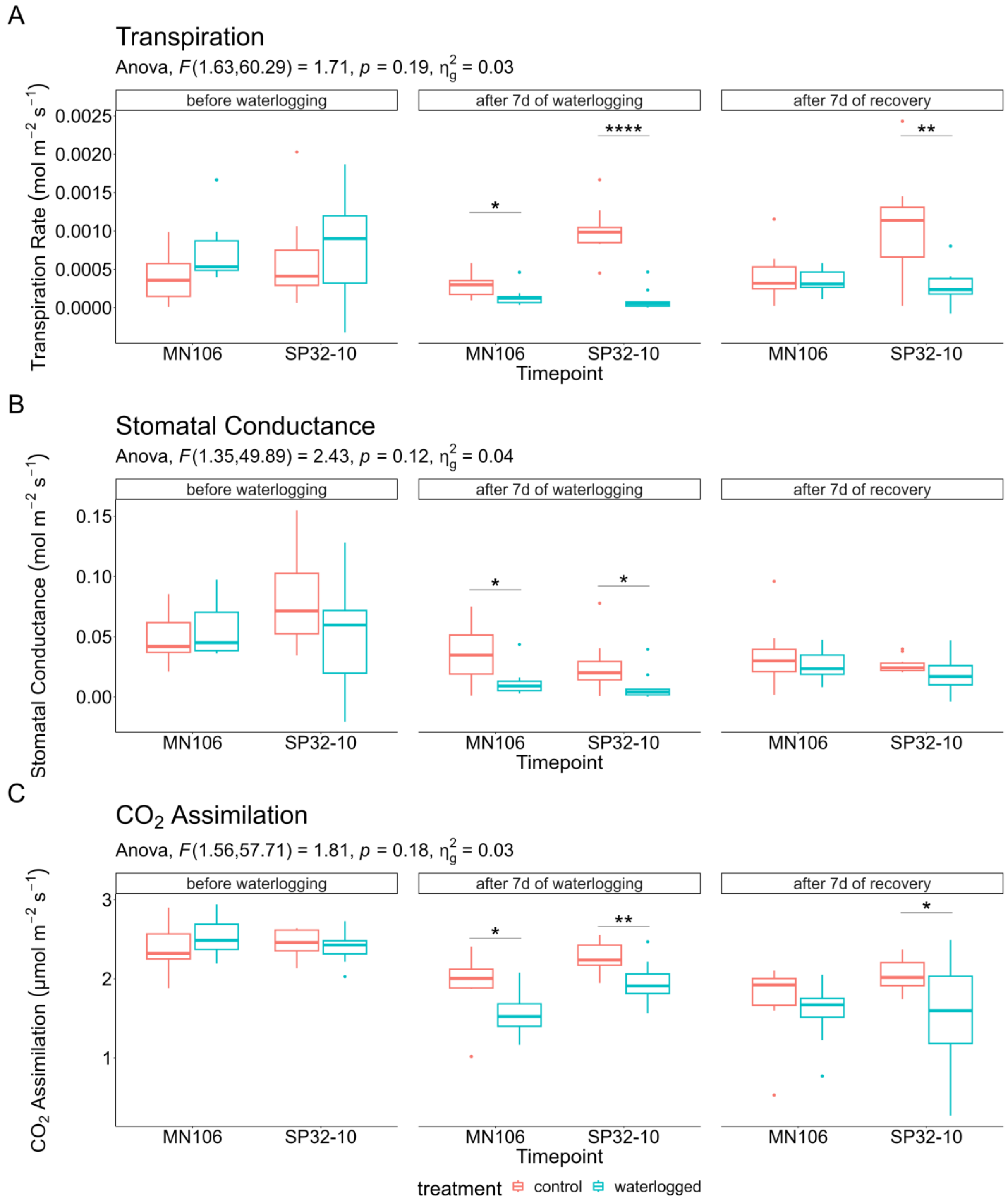

Supplementary Figure 2. Physiological responses before waterlogging, after 7 days of waterlogging, and after 7 days of recovery from waterlogging in the greenhouse experiment. A) Transpiration rate (log transformed), B) Stomatal conductance (log transformed), C) CO_2 assimilation (log transformed). * = p-value < 0.05, ** = p-value < 0.01, *** = p-value < 0.001, **** = p-value < 0.0001. Subtitle represents three-way mixed ANOVA result with F statistic, p-value, and generalized eta-squared.

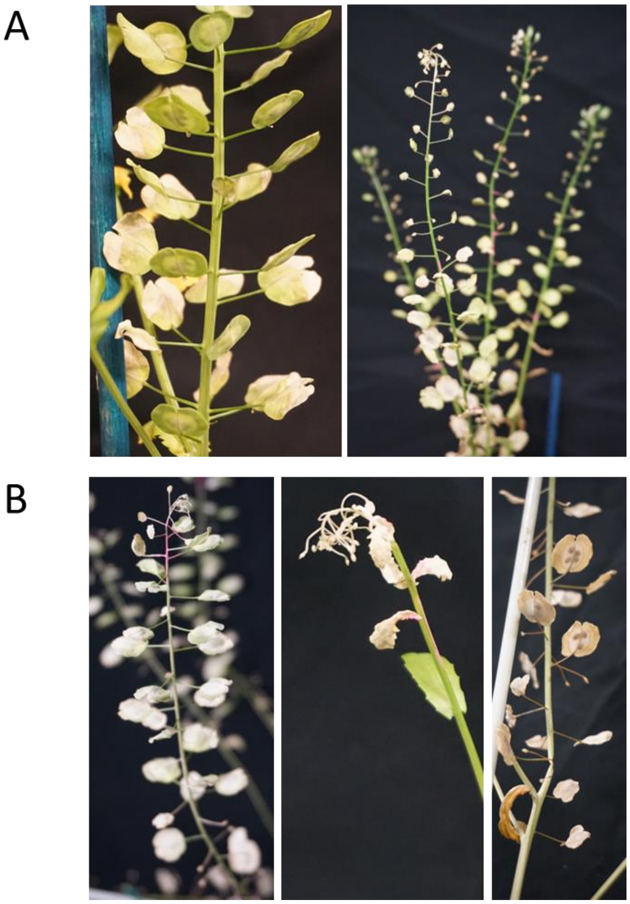

Supplementary Figure 3. Signs of early senescence on silicles and inflorescences after 1 week of recovery from waterlogging in the growth chamber experiment in A) MN106 and B) SP32-10.

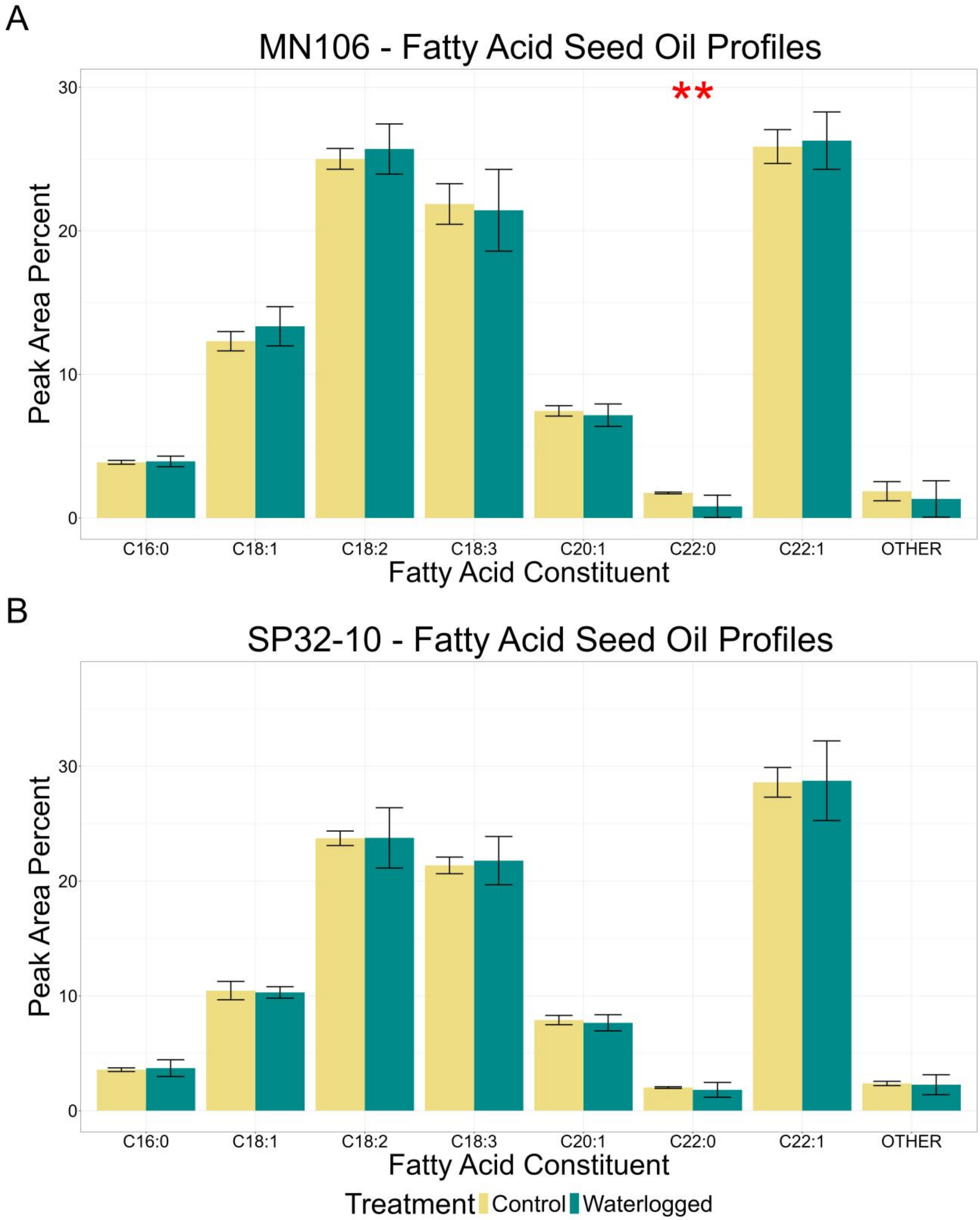

Supplementary Figure 4. Fatty acid oil profiles of mature seed after waterlogging treatment in the growth chamber experiment in A) MN106 and B) SP32-10. Fatty acid constituents are C16:0 = palmitic acid, C18:1 = oleic acid, C18:2 = linoleic acid, C18:3 = linolenic acid, C20:1 =

eicosenoic acid, C22:0 = behenic acid, C22:1 = erucic acid. Level of significance reported from Welch's Two Sample t-test. * = p-value < 0.05, ** = p-value < 0.01, ***=p-value < 0.001, bars denote standard deviation.

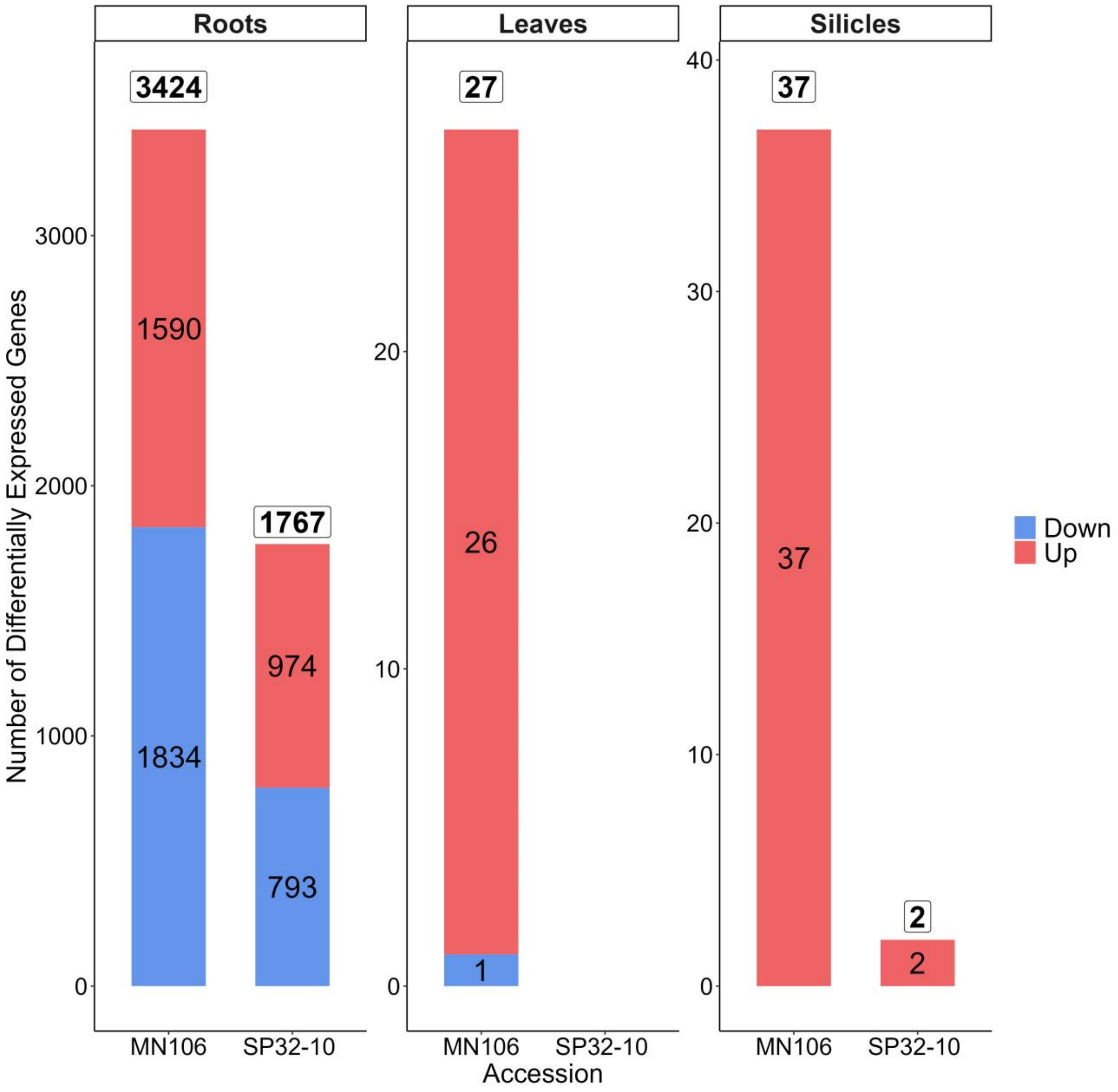

Supplementary Figure 5. Stacked bar plot representing the number of up and downregulated differentially expressed genes in roots, leaves, and silicles of MN106 and SP32-10 waterlogged compared to control samples.

Supplementary Figure 6. See attached file.
A dendrogram of the MENTOR output showing clusters of genes ordered by their functional or mechanistic relationships, along with a heatmap of log2FC values indicating upregulation (red) or downregulation (blue) in waterlogged vs control root samples. The clades outlined with a red box were chosen for discussion in the text.

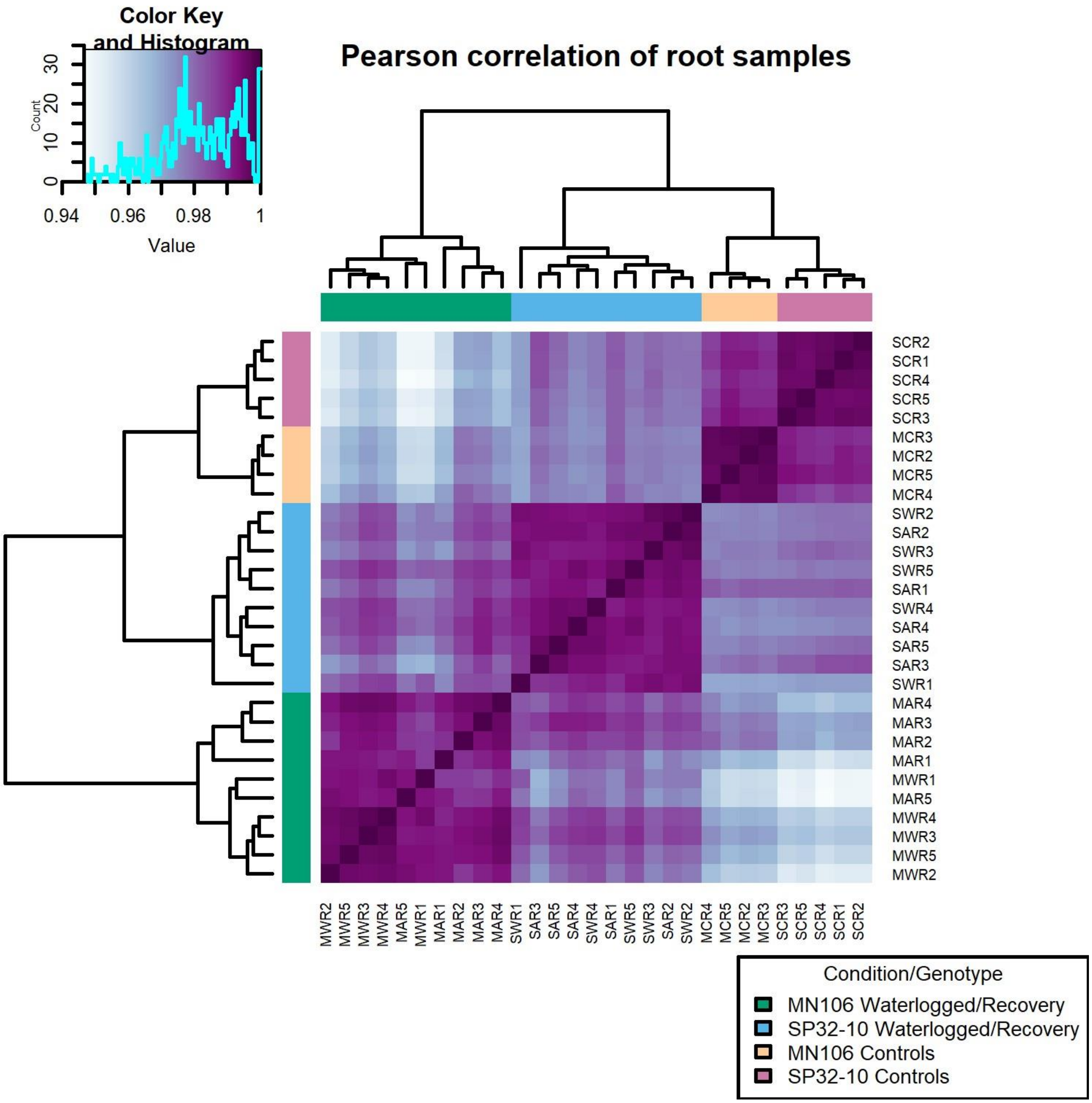

Supplementary Figure 7. Pearson correlation of all root samples with normalized counts as input. Biological replicates MWR1-5 and SWR1-5 indicate MN106 and SP32-10 waterlogged root samples, whereas MAR1-5 and SAR1-5 indicate recovery root samples. MCR1-5 and SCR1-5 are control root samples.
