# Supplementary material for: Morpho-physiological and transcriptomic responses of field pennycress to waterlogging
Source: Front Plant Sci. 2024 Dec 18;15:1478507. doi: 10.3389/fpls.2024.1478507 (PMC11688638; doi:10.3389/fpls.2024.1478507)
Supplement: Supplementary file 3 [file DataSheet3.pdf]

Supplementary Table 1. Means and standard deviations of morphological traits of waterlogged and control pennycress immediately after waterlogging in the growth chamber experiment.

	MN106			SP32-10		
	**Control**	**Waterlogged**	***P-value***	**Control**	**Waterlogged**	***P-value***
**Height (cm)**	64.42 ± 4.67	59.75 ± 9.40	0.421	70.92 ± 3.83	73.25 ± 2.27	0.235
**Branch #**	12.33 ± 4.03	12.67 ± 6.38	0.916	21 ± 4.20	19.5 ± 3.62	0.523
**Silicle #**	306.33 ± 67.5	271.67 ± 113.4	0.538	291.5 ± 69.6	282 ± 68.4	0.816
**Aborted Silicles (%)**	1.38 ± 1.74	3.76 ± 4.60	0.277	6.99 ± 3.08	13.54 ± 8.81	0.135

Sample size = 6. P-values derived from Welch’s t-test between treatments for each accession.

Supplementary Table 2. Mean values and standard deviations of the status of inflorescences before waterlogging, after waterlogging, and after 1 week of recovery in MN106 and SP32-10 in the growth chamber experiment.

	**MN106**			**SP32-10**		
	**Control**	**Waterlogged**	***P-value***	**Control**	**Waterlogged**	***P-value***
Before Waterlogging						
not yet flowering	0.39 ± 0.11	0.49 ± 0.15	0.199	0.47 ± 0.14	0.52 ± 0.11	0.546
flowering	0.56 ± 0.17	0.49 ± 0.17	0.487	0.53 ± 0.14	0.48 ± 0.11	0.546
done flowering	0.05 ± 0.12	0.02 ± 0.04	0.523	0	0	NA
dead	0	0	NA	0	0	NA
After Waterlogging						
not yet flowering	0.21 ± 0.11	0.28 ± 0.22	0.507	0.43 ± 0.08	0.26 ± 0.18	0.069
flowering	0.24 ± 0.24	0.15 ± 0.22	0.529	0.38 ± 0.18	0.18 ± 0.15	0.059
done flowering	0.38 ± 0.24	0.41 ± 0.12	0.821	0.19 ± 0.23	0.46 ± 0.22	0.063
dead	0.17 ± 0.12	0.16 ± 0.18	0.924	0	0.11 ± 0.09	0.023*
1 Week of Recovery						
not yet flowering	0.01 ± 0.03	0.08 ± 0.20	0.447	0.23 ± 0.20	0.10 ± 0.08	0.183
flowering	0.05 ± 0.09	0.08 ± 0.11	0.668	0.02 ± 0.03	0	0.211
done flowering	0.57 ± 0.13	0.48 ± 0.13	0.269	0.71 ± 0.18	0.70 ± 0.13	0.925
dead	0.36 ± 0.15	0.35 ± 0.23	0.936	0.04 ± 0.09	0.19 ± 0.17	0.083

Means were determined by the number of inflorescences for each status (not yet flowering, flowering, done flowering, dead) divided by the total number of inflorescences on the plant. Sample size = 6. Asterisk denotes statistical significance of < 0.05.

Supplementary Table 3. Means and standard deviations of morphological traits of waterlogged and control pennycress after 1 and 2 weeks of recovery from waterlogging in the growth chamber experiment.

	MN106			SP32-10		
	**Control**	**Waterlogged**	***P-value***	**Control**	**Waterlogged**	***P-value***
1 week						
**Height (cm)**	64.4 ± 4.67	60.5 ± 9.22	0.572	71.4 ± 4.36	74.5 ± 3.45	0.206
**Branch #**	12.3 ± 4.03	13.5 ± 5.72	0.693	24.5 ± 7.09	20.8 ± 3.82	0.298
**Silicle #**	335.2 ± 73.5	282.2 ± 111.8	0.358	374.3 ± 58.0	326.3 ± 57.8	0.182
**Senesced Silicles (%)**	0	1.70 ± 3.9	0.176	0	18.8 ± 30.7	0.010**
**Aborted Silicles (%)**	2.76 ± 3.24	5.71 ± 6.85	0.371	21.8 ± 7.82	21.5 ± 6.19	0.945
2 weeks						
**Silicle #**	336.3 ± 74.9	291.5 ± 121.4	0.463	392.7 ± 78.7	333.3 ± 62.3	0.394
**Senesced Silicles (%)**	4.53 ± 6.0	24.7 ± 25.3	0.111	7.03 ± 5.44	30.1 ± 34.4	0.009**

Sample size = 6. Asterisk denotes statistical significance of < 0.05. P-values derived from Welch's t-test between treatments for each accession.

Supplementary Table 4. Means and standard deviations of morphological traits of waterlogged and control plants at the time of harvest in the growth chamber experiment.

	MN106			SP32-10		
	**Control**	**Waterlogged**	***P-value***	**Control**	**Waterlogged**	***P-value***
**Height (cm)**	64.8 ± 4.75	59.8 ± 10.2	0.465	71.4 ± 4.36	74.5 ± 3.45	0.206
**Reproductive Height (cm)**	30.3 ± 4.34	27.5 ± 5.14	0.341	31.3 ± 4.37	33 ± 3.29	0.474
**Primary Branch #**	5.17 ± 1.33	4.5 ± 2.17	0.538	7.83 ± 1.94	7 ± 2.53	0.540
**Branch #**	12.3 ± 4.03	13.5 ± 5.72	0.693	24.5 ± 7.09	20.8 ± 3.82	0.298
**Silicle #**	339.3 ± 74.9	306.3 ± 133.6	0.612	401.7 ± 72.3	352.8 ± 61.1	0.394
**Aborted Silicles (%)**	16.9 ± 3.1	25.1 ± 9.39	0.091	35.5 ± 6.17	44.5 ± 3.23	0.014*
**Maturity**	45.7 ± 1.86	44.2 ± 4.02	0.434	42.7 ± 1.03	41.2 ± 4.49	0.458
**Shoot Dry Weight (g)**	4.27 ± 1.05	3.3 ± 1.20	0.169	3.59 ± 0.46	3.09 ± 0.73	0.299
**Total Seed Count**	1588.2 ± 486.3	1131.2 ± 500.2	0.140	1342.8 ± 217.5	948.7 ± 223.9	0.011*
**Total Seed Weight (g)**	1.35 ± 0.34	1.01 ± 0.51	0.201	1.23 ± 0.19	0.89 ± 0.31	0.048*
**Thousand Seed Weight (g)**	0.87 ± 0.06	0.86 ± 0.14	0.876	0.92 ± 0.07	0.93 ± 0.23	0.310
**Single Seed Weight (mg)**	0.98 ± 0.10	0.74 ± 0.17	0.018*	0.95 ± 0.46	0.91 ± 0.27	0.864
**Oil Content (% DWB)**	32.2 ± 1.12	28.8 ± 2.60	0.004**	29.5 ± 2.21	29 ± 3.31	0.675

Sample size = 6. Asterisk denotes statistical significance of < 0.05. DWB = dry weight basis. P-values derived from Welch's t-test between treatments for each accession.

Supplementary Table 5. Functional enrichment categories from an over-representation analysis of the unique upregulated and downregulated differentially expressed genes (DEGs) in MN-7WL and SP-7WL.

**Upregulated unique DEG functions**		**Downregulated unique DEG functions**	
**MN-7WL**	**SP-7WL**	**MN-7WL**	**SP-7WL**
**GO Terms**		**GO Terms**	
response to decreased oxygen levels	None	secondary metabolite biosynthetic process	glycosinolate metabolic process
small molecule catabolic process		inorganic ion homeostasis	secondary metabolite biosynthetic process
response to hypoxia		phenylpropanoid metabolic process	cell wall biogenesis
response to salicylic acid		intracellular iron ion homeostasis	oxidoreductase activity
organic acid catabolic process		heme binding	
carboxylic acid catabolic process		salt transmembrane transporter activity	
single-stranded RNA binding		oxidoreductase activity	
flavin adenine dinucleotide binding		molecular transducer activity	
carbohydrate binding		metal ion transport	
		tetrapyrrole binding	
		signaling receptor activity	
		tropism	
		pattern specification process	
		root epidermal cell differentiation	
		antioxidant activity	
		lignin metabolic process	
		trichoblast differentiation	
		cell wall biogenesis and organization	
		meristem structural organization	
		regionalization	
		hydrolase activity	
		transmembrane receptor protein kinase activity	

		response to gravity	
		DNA-binding transcription factor activity, RNA polymerase II-specific	
		polysaccharide catabolic process	
		response to starvation	
		lipase activity	
		channel activity	
		transferase activity	
		alpha-amino acid metabolic process	
		meristem maintenance	
		trichoblast maturation	
		xylem and phloem pattern formation	
**KEGG Terms**		**KEGG Terms**	
Glycerolipid metabolism	Carbon metabolism	Phenylpropanoid biosynthesis	None
Carbon metabolism	Biosynthesis of amino acids	Starch and sucrose metabolism	
Glycolysis / Gluconeogenesis	Glycerolipid metabolism		
Phenylpropanoid biosynthesis	Glycolysis / Gluconeogenesis		
Biosynthesis of amino acids	Phenylpropanoid biosynthesis		
