# Supplementary material for: Morpho-physiological and transcriptomic responses of field pennycress to waterlogging
Source: Front Plant Sci. 2024 Dec 18;15:1478507. doi: 10.3389/fpls.2024.1478507 (PMC11688638; doi:10.3389/fpls.2024.1478507)
Supplement: Supplementary file 4 [file DataSheet4.pdf]

Supplementary Methods

Total Seed Oil Content

Total oil content was determined by nondestructive TD-NMR on 450 mg samples of whole pennycress seed. TD-NMR was performed on the samples and standards with a Bruker Minispec MQ40 with a 39.95 MHz NMR frequency and 40°C magnet temperature. The 90° and 180° pulse lengths were 11.48 µs and 22.98 µs respectively. A 23° detection angle, gain of 44 dB, pulse attenuation of 15 dB, recycle delay of 2 s, window 1 of 0.055 ms, window 2 (τ_{ε}) of 7 ms, and 724 magnetic field steps were set to employ a Hahn spin echo effect for signal collection. Each sample and standard analysis employed 16 scans at a receiver gain of 59. The percent oil for each sample was calculated using the Bruker Oil and Moisture in Seeds application in minispec Plus version 7.0.0 software.

A percent oil calibration curve was constructed within the Bruker software by measuring the signal for six oil standards between 20% -40% oil concentration based on a 450 mg sample. Crude pennycress oil in six amounts ranging from 90 mg to 180 mg was carefully dropped down six 10 mm flat bottom NMR tubes containing a piece of kimwipe in the bottom. After the oil standards were tempered at 40°C for 30 minutes, they were scanned in the MQ40 in the calibration mode to create a signal vs concentration calibration curve for percent oil.

To determine percent oil in seeds from the sample plots, an analytical balance was used to weigh 450 mg of seed from each plot into 10 mm flat bottom NMR tubes. After tempering the samples at 40°C for 30 minutes, they were scanned in the MQ40 and the percent oil was calculated using our calibration curves and the Bruker Oil and Moisture in Seeds application in minispec Plus software. The results from minispec Plus were exported to an Excel file. Using Excel, the percent oil on a dry weight basis was calculated by dividing the milligrams of oil in each sample by the dry weight of each sample and multiplying by 100.

Fatty Acid Seed Oil Composition

For gas chromatography (GC) analysis of oil components, medium-chain triglycerides were extracted and converted to fatty acid methyl esters (FAMEs) through a transesterification reaction. A Mettler Toledo analytical balance was used to weigh 100 mg of seed from each plot into 20 mL glass scintillation vials. To each sample, 5 mL of 0.25 M sodium methoxide in methanol was added, and an Ingenieurburo CAT X 120 homogenizer was used to thoroughly grind the seed. The vials were capped and placed in an oven at 60°C. After 30 minutes, the vials were removed and cooled to room temperature. To each vial, 2 mL of saturated sodium chloride in distilled water and 5 mL of hexane were added. The vials were shaken and two separate layers developed. Approximately 1.5 mL of the top layer which was comprised of hexane with FAMEs was pipetted into a GC autosampler vial for GC analysis.

The fatty acid methyl esters were analyzed using an Agilent 6890 gas chromatograph with a 7683 autosampler and flame ionization detector (FID). A Supelco 2380 30 m x 0.25 mm x 0.2 µm film thickness column was used. Ultra-high purity helium was the carrier gas with a constant pressure of 20.00 psi. A 1 µL injection was used with a split ratio of 50:1. Split flow and total flow were 58.3 mL/min and 62.2 mL/min. For the detector, ultra-high purity hydrogen and air

were used in the FID with flow rates of 40.0 mL/min and 450.0 mL/min respectively. The injector and detector temperatures were set at 265°C and 250°C respectively. The oven temperature was set initially at 170°C and ramped at 4°C/min to 190°C then ramped at 30°C/min to 265°C and held for 2.5 min. NuChek standard 17A and Supelco 37 component FAME standard were run to match retention times. Data was collected and the area percents for the FAMEs were exported into an Excel file using Agilent OpenLab CDS ChemStation Edition revision C.01.10 software.
