# Supplementary material for: Morpho-physiological and transcriptomic responses of field pennycress to waterlogging
Source: Front Plant Sci. 2024 Dec 18;15:1478507. doi: 10.3389/fpls.2024.1478507 (PMC11688638; doi:10.3389/fpls.2024.1478507)

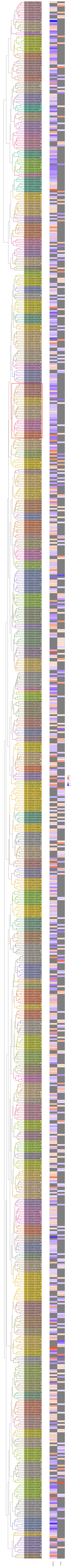

Supplement: Supplementary file 2 [file DataSheet2.pdf]
